# tRNA-modifying enzymes in bacterial stress adaptation

**DOI:** 10.1098/rsob.250194

**Published:** 2025-10-08

**Authors:** Louna Fruchard, Claudia Salinas, Andre Carvalho, Zeynep Baharoglu

**Affiliations:** ^1^ Epitranscriptomic and Translational Responses to Antibacterial Stress Team, Expression Génétique Microbienne, CNRS UMR8261, Institut Pasteur, Université Paris Cité, Institut de Biologie Physico-Chimique, Paris, France; ^2^ Sorbonne Universite, Collège Doctoral, F-75005, Paris, France; ^3^ Ecole Doctorale 474 FIRE, Universite Paris Cite, Paris, France; ^4^ Institut Pasteur, Université Paris Cité, Unité Plasticité du Génome Bactérien, 75015, Paris, France; ^5^ Complutense University of Madrid Faculty of Veterinary, Madrid, Community of Madrid, Spain

**Keywords:** epitranscriptomics, tRNA modifications, bacterial stress responses, oxidative stress, modification tunable transcipts, transfer RNAs, tRNA-modifying enzymes, antibiotic stress, bacterial adaptive responses, selective translation

## Abstract

Transfer RNAs (tRNAs) and their modifications are central to bacterial translation and physiology, yet their roles in stress adaptation remain underexplored. While extensively studied in eukaryotes, and linked to diseases, bacterial tRNA modifications are only recently gaining attention. This review highlights emerging insights into how tRNA modifications and associated enzymes contribute to bacterial survival under oxidative and antibiotic stresses, both disrupting proteostasis. We examine the environmental and physiological stresses bacteria encounter, focussing on reactive oxygen species and sub-lethal antibiotic exposure. These stresses challenge proteome integrity and trigger adaptive responses involving key stress regulators. We explore the expanding field of bacterial epitranscriptomics, detailing the diversity, dynamics and structural impact of tRNA modifications, and how they influence selective translation. Central to this is the concept of modification tunable transcripts, linking specific codon usage patterns to stress-responsive translation reprogramming. Beyond their catalytic roles, tRNA-modifying enzymes also have additional functions. We discuss this dual functionality and its broader implications for bacterial adaptability. By integrating recent technological advances and conceptual models, this review underscores the potential of targeting tRNA modifications as a novel strategy to combat bacterial pathogenicity and antibiotic resistance. With many aspects still unresolved, the study of bacterial tRNA modifications promises rich opportunities for discovery and therapeutic innovation.

**Figure:**
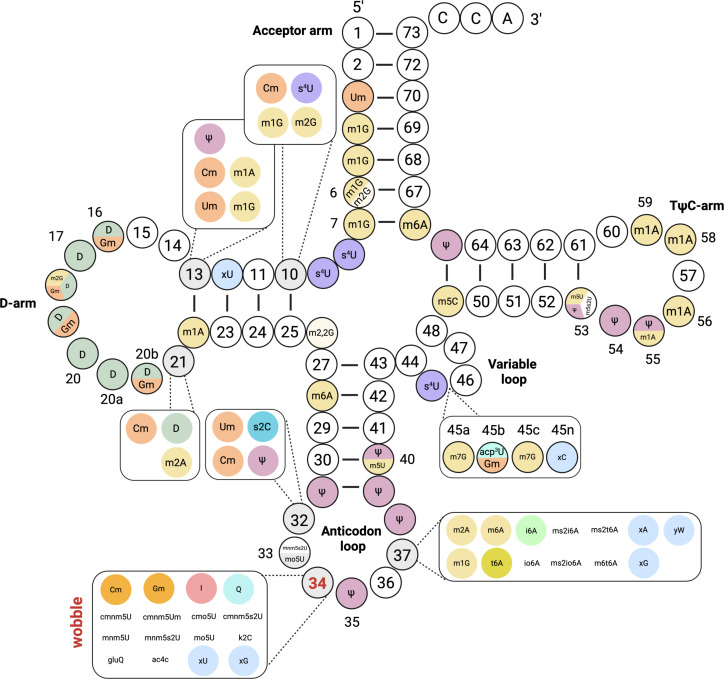

## 1. Introduction

Transfer RNAs (tRNAs) are vital for translation and bacterial function, with tRNA modifying enzymes crucially regulating key cellular processes. While extensively studied in humans, where tRNA modifications link to diseases like cancer, diabetes and intellectual disability, their roles in bacteria remain underexplored. Understanding bacterial tRNA modifications is critical amid the antimicrobial resistance crisis, as bacteria rapidly adapt through conserved mechanisms and serve as ideal models for research.

Prokaryotes thrive in diverse, often extreme environments, constantly facing abiotic stresses (temperature, pH, oxidative stress, nutrient limitation) and ecological competition, including antibiotics. Past reviews have detailed tRNA modification diversity and regulation [1–7], with growing bacterial-focussed research since 2000.

Reactive oxygen species (ROS) and sub-lethal antibiotic exposure are particularly important in this context, as they represent common and often simultaneous challenges to bacterial survival. ROS can arise from environmental sources or as a by-product of metabolism, damaging DNA, proteins and lipids, while many antibiotics, including aminoglycosides, both induce ROS production and disrupt proteostasis. These overlapping stresses require coordinated responses that maintain translation fidelity, selectively adjust protein synthesis and stabilize the proteome under damage pressure.

tRNA modifications are increasingly recognized as key regulators in this adaptive process. Under stress, changes in tRNA modification patterns can bias translation towards proteins that promote survival, while limiting synthesis of non-essential or stress-sensitive proteins. This selective translation reprogramming provides a powerful, rapid and reversible means for bacteria to remodel their proteome in response to ROS and antibiotic stress.

A review centred on tRNA modifications in the context of ROS and antibiotic stress is timely because it links two major, interconnected stressors with an adaptive molecular system that is both highly conserved and functionally versatile. By focussing on how tRNA modifications and their enzymes influence bacterial responses to oxidative and antibiotic stress, this work aims to clarify their contribution to survival strategies, highlight potential vulnerabilities and point towards novel approaches for tackling antibiotic resistance.

After outlining the link between these stresses and bacterial physiology, we examine tRNA-modifying enzymes and their vital role in stress response and adaptation. Their diversity, specific impacts on proteins and pathways, and potential regulation by stress conditions make them compelling targets for further study.

## 2. Bacterial life is stressful: stress in the environment

### 2.1. Oxidative challenges throughout *the bacterial* life cycle

#### 2.1.1. Oxygen: sustainer and threat

**Figure 1.**
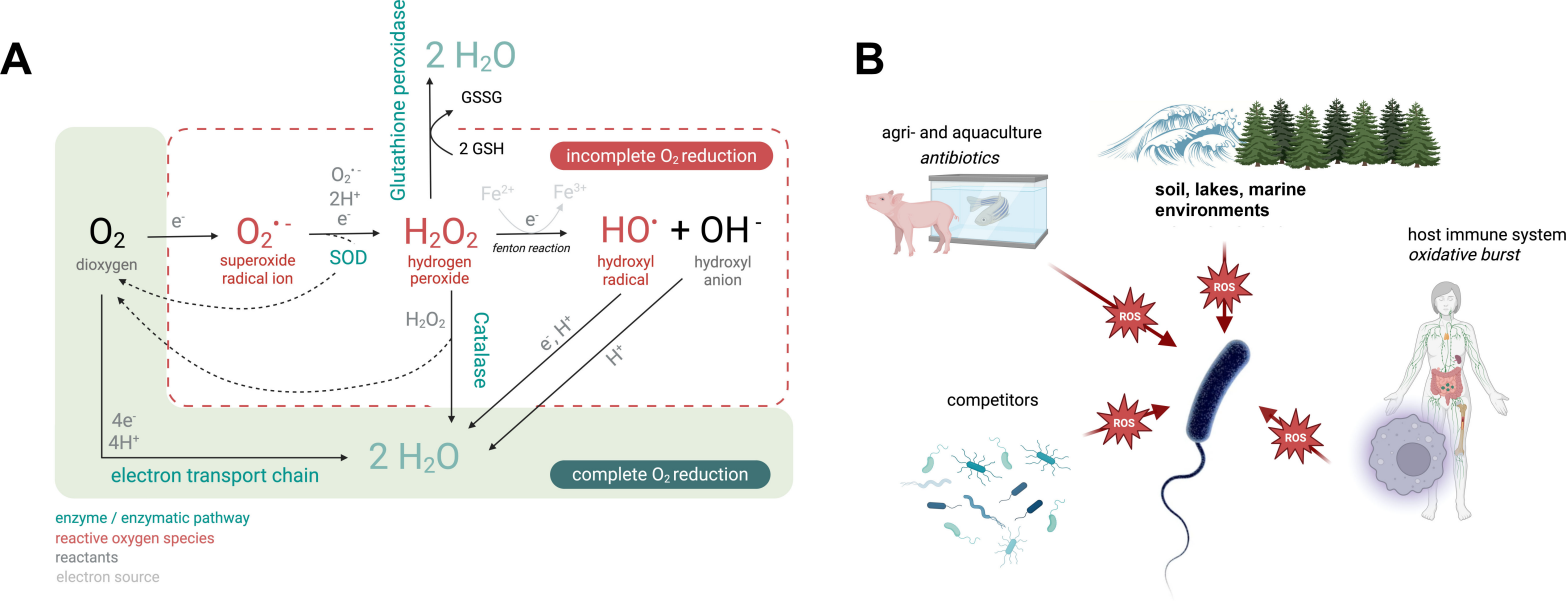
(A) Sequential one-electron and direct four-electron reduction of O_2_ to water. The successive reduction of O_2_ by single electrons is composed of four reduction steps resulting in intermediate ROS (in red). H_2_O_2_ reduction to HO^•^ occurs via the Fenton reaction, necessitating an iron (Fe) catalyst, Fe^2+^, which will be oxidized into Fe^3+^. The final step is the addition of an electron and a H^+^ to HO^•^ to obtain water. HO^•^, O_2_^•−^ and H_2_O_2_, classified as ROS, are more likely to interact with biomolecules than O_2_. Singlet molecular oxygen (^1^O_2_), the last of the four major ROS (not represented here), is highly toxic to organisms, as it also reacts with most organic molecules, including deoxyribonucleic acid (DNA), ribonucleic acid (RNA), proteins, lipids and thus alters cellular functions [9]. SOD stands for superoxide dismutase. (B) The diverse ROS-generating conditions surrounding bacteria throughout their lifecycle.

Although current concerns focus on carbon dioxide (CO_2_), the major challenge facing organisms two billion years ago was dioxygen (O_2_) [8]. While many bacteria today rely on oxygen for energy production and for the synthesis of various essential metabolites, excessive oxygen levels are toxic. ROS are generated through spontaneous or enzyme-mediated reduction of molecular oxygen, producing superoxide (O_₂_^•⁻^), hydrogen peroxide (H_₂_O_₂_), hydroxyl radical (HO^•^) and hydroxyl anion (OH^⁻^) (figure 1A). Their reactivity follows the order: HO^•^ > ^¹^O_₂_ > O_₂_^•⁻^ > H_₂_O_₂_ [10]. To survive, organisms have evolved mechanisms to maintain redox balance and mitigate oxygen-induced damage [11]. Redox balance, the equilibrium between oxidants and antioxidants, is essential for cell function. When ROS exceed antioxidant capacity, oxidative stress (OS) occurs, a concept introduced by Sies in the 1990s [12]. Figure 1B illustrates various sources of ROS that bacteria encounter throughout their lifecycle.

#### 2.1.2. Respiration

Dioxygen is a potent electron acceptor that diffuses freely across membranes, preventing cells from lowering internal O_₂_ levels below ambient concentrations. Studies in bacteria show endogenous ROS production; for example, *Escherichia coli* generates 10−15 µM H_₂_O_₂_ per second under air-saturated conditions [13].

#### 2.1.3. The aquatic environment

Natural water contains 20−1400 nM H_₂_O_₂_ [14], primarily from solar radiation, with concentrations fluctuating in sync with the day–night UV cycle [15]. ROS levels are higher on sunlit surfaces due to photochemical production. Rainfall can increase ROS over 10-fold, especially at the ocean surface and down to 50 m. Anthropogenic pollution further elevates H_₂_O_₂_, while cyanobacterial reservoirs contribute to oxidative stress through photosynthesis.

#### 2.1.4. The host

During infections, various host immune cells undergo an ‘oxidative burst’ or ‘respiratory burst’ [16]. This pathogen-fighting process relies on the release of ROS [17], enabling macrophages to oxidatively damage and degrade engulfed bacteria, key to innate immunity. Supporting this, and as an example, stool samples [18] and duodenal biopsies [19] from cholera patients show elevated ROS levels compared to healthy individuals, indicating that *Vibrio cholerae* encounters toxic ROS in the host.

#### 2.1.5. Other reactive oxygen species-generating factors

Microorganisms can produce and secrete ROS to eliminate competitors in nutrient-limited environments, gaining a competitive advantage [20]. This may result from long-term co-evolution between closely related species.

Finally, and as detailed below, antibiotic exposure can lead to ROS accumulation.

### 2.2. Antibiotic challenge in bacterial environments

#### 2.2.1. Antibiotics in the environment

**Figure 2.**
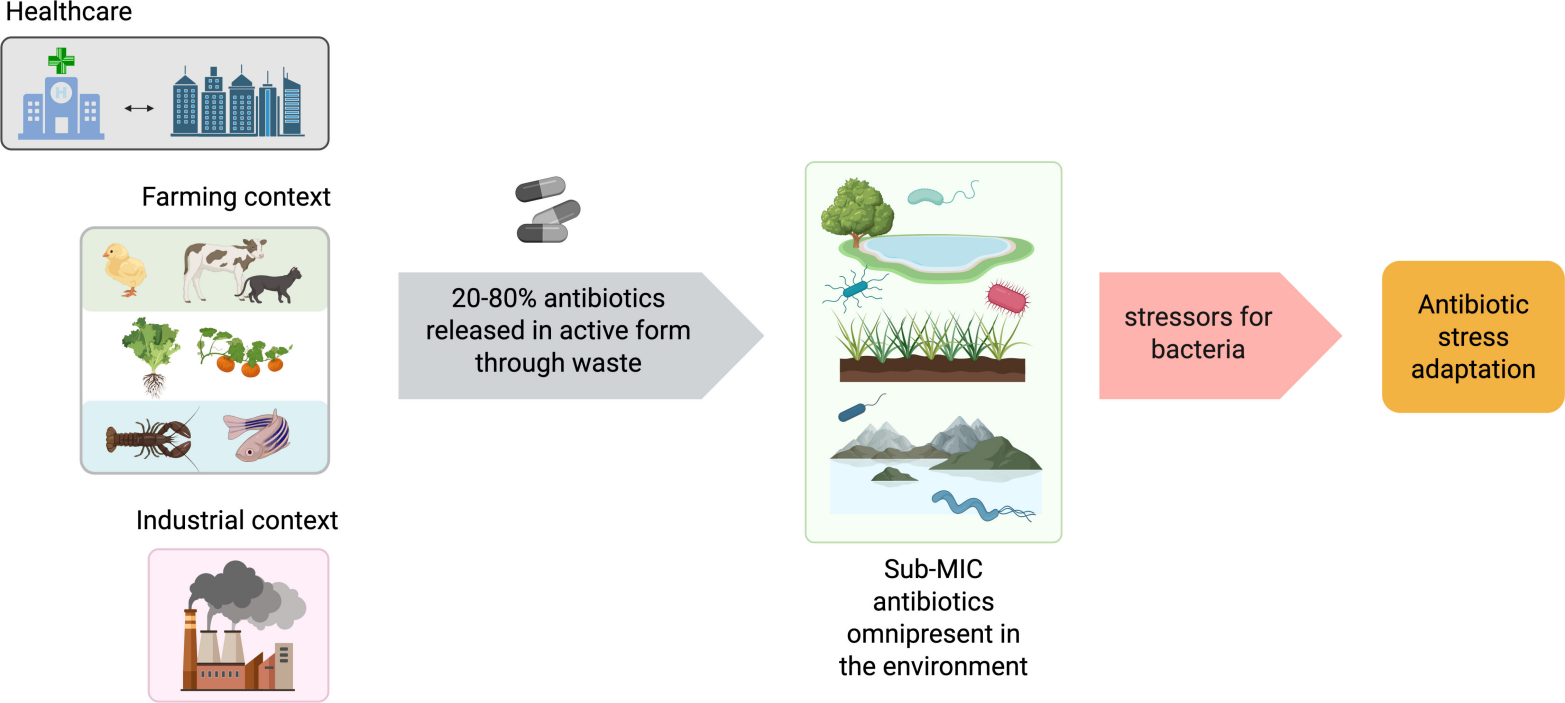
Environmental dissemination and impact of antibiotics on bacterial stress and adaptation (adapted from [28]). Circulation of antibiotics from major anthropogenic sources into natural ecosystems lead to their accumulation in soil, freshwater, and marine environments. This widespread sublethal pressure imposes continuous selective and physiological pressures on bacteria, promote adaptive responses and thus contribute to AMR’s emergence and spread. Antibiotic stress adaptation has been described to be through transcriptional [29–32] as well as post-transcriptional regulation (reviewed here).

Antibiotics, whether natural or synthetic, inhibit bacterial growth (bacteriostatic) or kill bacteria (bactericidal), targeting pathogens with minimal host damage. Since the discovery of the first antibiotic, it became clear that many microbes produce such compounds to inhibit competitors, making their isolation vital for treating infections. New classes and synthetic derivatives revolutionized twentieth-century medicine. Antibiotics were also used as agricultural growth promoters [21] until banned in Europe in 2006 [22]. Residues from farming and human use enter the environment through food, urine and wastewater, where treatment plants often fail to fully remove them [23]. These environmental antibiotic gradients [21,23–25] drive the emergence and spread of resistant strains [21,26,27] (figure 2).

#### 2.2.2. The underestimated importance of low doses of antibiotics

Sub-MIC (sub-inhibitory concentrations) of antibiotics alter bacterial transcriptomes and proteomes [29,33], affecting both targeted and unrelated pathways (for a review [28]), sometimes with effects opposite to those at high doses [34]. They can also trigger ROS accumulation and oxidative stress [35,36]. Understanding these responses is essential for addressing resistance.

## 3. Epitranscriptomic adaptation to stress from a tRNA perspective

### 3.1. The tRNA function, structure and biogenesis

**Figure 3.**
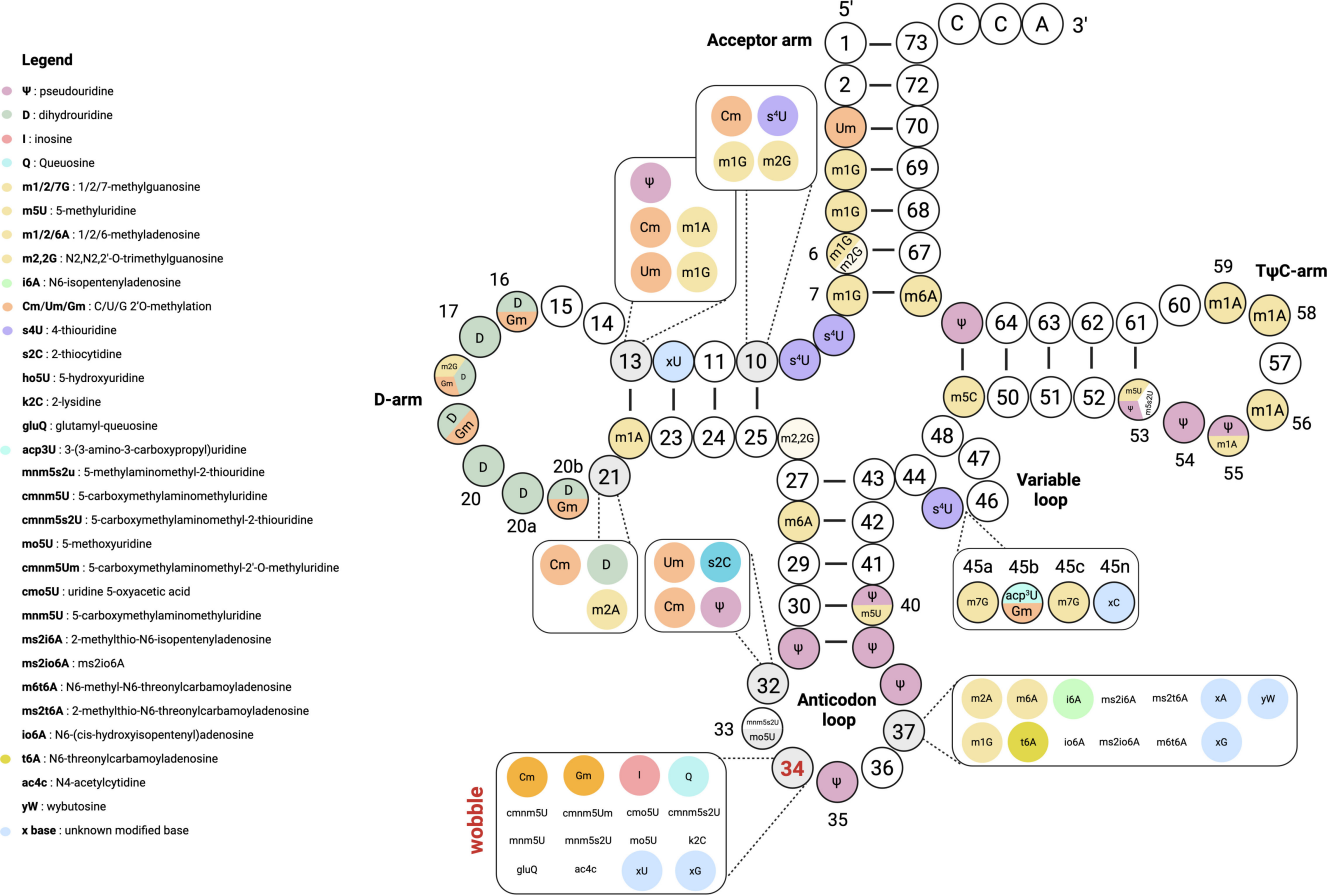
tRNA modifications in bacteria. In the schematic representation of the tRNA molecule, each nucleotide position is labelled with the modification(s) identified in bacteria (source: MODOMICS [38]) . Next to each modification, the associated catalysing enzyme is indicated. In cases where the modifying enzyme has not been identified yet, ‘unknown’ is written. We note that methods employed for modified residues identification do not detect all modifications, and therefore, the list provided is not exhaustive. For instance, 2-thiouridine (s2U), among others, is not included in this representation. (Included species: *Azospirillum lipoferum, Bacillus subtilis, Escherichia coli, Geobacillus stearothermophilus, Lactococcus lactis, Mycobacterium smegmatis, Mycoplasma capricolum, M. mycoides, Rhodospirillum rubrum, Salmonella typhimurium, Spiroplasma citri, Staphylococcus epidermidis, Streptomyces coelicolor A3(2), S. griseus, Synechococcus elongatus PCC 6301, Sy.* sp*. PCC 7002, Synechocystis* sp*., Thermus aquaticus, T. thermophilus*.)

tRNAs are short non-coding RNAs (approx. 70−100 nucleotides) essential for translation, delivering amino acids to the ribosome to ensure proteins match their genes (for a review: [37]). They fold into a conserved cloverleaf secondary structure and three-dimensional L-shape (figure 3), with key regions including the acceptor stem (amino acid carrier), D-arm, anticodon arm (with the anticodon triplet at positions 34−36), variable loop and TψC-arm. The D- and TψC-arms interact to form the hydrophobic elbow. The anticodon arm consists of a five-base pair stem and the anticodon loop (ACL), containing the triplet anticodon that pairs with mRNA codons. The anticodon’s wobble position (nucleotide 34) allows flexible base pairing.

tRNAs are often transcribed as polycistronic RNAs and processed into mature forms by cleavage, trimming and modifications [39]. The final step is aminoacylation, attaching the correct amino acid via aminoacyl-tRNA synthetases.

Bacterial adaptability stems from genetic variation through mutation, horizontal gene transfer and epigenetics, driving phenotypic diversity and survival under stress. Gene expression is regulated at multiple levels, with RNA modification enzymes playing a key role in shaping the proteome. The next section explores tRNA modification genes in bacterial adaptation.

### 3.2. tRNA modifications

#### 3.2.1. Diversity and roles

Before the mid-twentieth century, nucleic acids were thought to contain only four bases (A, T/U, G, C). It is now known that RNAs are highly modified molecules [40]. Over 170 RNA modifications have been identified across all three domains of life and viruses [38], with more than 110 found in tRNAs, making them the most modified RNA class. In bacteria, 2−15% of tRNA bases are modified, with a median of eight modifications per molecule [38].

Most tRNA modifications occur in the anticodon loop (ACL), D-loop and TψC-loop, with positions 34 and 37 showing the highest diversity [38] (see review on tRNA modification circuits in the anticodon loop [41]). These modifications, part of the epitranscriptome, are added by specific enzymes and range from simple (e.g. methylation, thiolation) to complex (e.g. queuosine). They can affect the ribose or nucleobase, influencing tRNA structure [42] and degradation [43], from subtle to major conformational changes [44] (figure 3). The MODOMICS database [38] catalogues these modifications, their enzymes and biosynthetic pathways. The (non-exhaustive) diversity of tRNA modifications identified in bacteria is shown in figure 3.

To date, the roles of tRNA modifications appear to depend largely on their specific location within the molecule [7]. Modifications at the anticodon loop (ACL) have been reported to influence aminoacylation rate [45–47] and reliability [48], codon decoding, translation speed, accuracy [49], reading-frame maintenance [50] and tRNA fragment (tRF) biogenesis [51]. Modifications outside the ACL are generally thought to have minimal direct impact on codon–anticodon pairing. However, they can influence various aspects of tRNA function, such as ribosome affinity, structural stability and maturation, ultimately affecting protein synthesis [52,53].

#### 3.2.2. tRNA modifications are dynamic

tRNA modifications were once seen as static, but are now known to be dynamically regulated. Bacterial tRNA modification profiles can change rapidly in response to internal or external cues, such as growth phase [54], rate [55], stress [56,57], nutrient levels [58], oxygen [56,59] and temperature [60,61]. tRNA modification levels can also respond to phage infection, boosting phage gene expression [62]. This is achieved through shifts in expression of modifying enzymes, tRNA synthesis or degradation and demodifying enzyme activity. Together, these mechanisms allow for dynamic epitranscriptomic regulation. Technological advances have helped track such changes under stress [63–65], but the specific roles of dynamic tRNA modifications in bacterial adaptation are still not fully understood.

Beyond RNA-modifying enzymes (*writers*), cells also use *erasers* (which remove modifications) [66,67] and *readers* (which recognize modifications), playing a crucial role in interpreting them and facilitating information transmission within the cell; reviewed in [68]. Without erasers, bacteria would rely on slow tRNA turnover to shift modification profiles, an impractical delay, given tRNAs’ high *in vivo* stability [69]. Methylations are the most studied reversible RNA modifications, especially in eukaryotes, where several demethylases have been characterized [70–75]. In contrast, few bacterial tRNA demodifying enzymes have been identified. The only known examples are Rmd enzymes, which remove N¹-methyladenosine in *Streptomyces venezuelae* [67], and RudS enzymes, proposed to reverse 4-thiouridine [66]. These findings suggest demodifying activity also exists in bacteria and more such enzymes remain to be discovered.

Notably, some tRNA modifications depend on the presence of others. While certain enzymes act directly on unmodified tRNAs, others require prior modifications to function. These interdependencies form ‘modification circuits’, especially in the anticodon loop [41,76,77], coordinating the timing and structure of tRNA decoration.

#### 3.2.3. Modifications can impact tRNA structure and stability

Post-transcriptional tRNA modifications are essential for forming the thrree-dimensional L-shape through D- and T-arm interactions. Pseudouridine (Ψ), the most abundant RNA modification, stabilizes the T-arm and enhances D–T arm binding (a review [78]). TruB-mediated Ψ55 is crucial for bacterial adaptation to temperature shifts, aiding *E. coli* in heat shock survival [79] and supporting cold-shock growth in *Thermus thermophilus* [80].

Dihydrouridine (D), produced by dihydrouridine synthases (Dus), is found mainly in the D-loop and varies by tRNA, species, and environment. In *E. coli*, DusA, DusB and DusC generate D at positions 16, 17, 20 20a. While once thought to stabilize the D-stem-loop structure, D is now known to increase flexibility and conformational dynamics [81].

These modifications fine-tune tRNA stability and act as quality control signals. Hypomodification, especially outside the ACL, can trigger targeted tRNA decay and reduce aminoacylation [43,82]. They also regulate tRNA cleavage [83].

#### 3.2.4. Codon–anticodon base pairing: the wobble pairing hypothesis

In 1966, Francis Crick proposed the wobble hypothesis, suggesting that the anticodon’s 34th nucleotide (wobble position) can form non-canonical base pairs with the third codon base [84]. Unlike strict Watson–Crick–Franklin pairing at positions one and two, wobble allows flexible pairing, such as modified U pairing with G and inosine pairing with U, C or A [85]. These modifications enable one tRNA anticodon to recognize multiple codons, explaining genetic code degeneracy and why organisms like *E. coli* have fewer tRNAs: 35, than codons: 61. Only methionine and tryptophan have unique codons, and 30−40% of decoding relies on wobble pairing [85]. Certain base modifications can expand the codon recognition range of a specific anticodon [86–89], while others can inhibit or restrict wobble pairing [90,91].

In 1982, Yarus introduced the extended anticodon concept, emphasizing the role of nucleotides around the anticodon and their modifications in codon recognition [92]. Position 37, a frequently modified purine adjacent to the anticodon, stabilizes codon–anticodon pairing, prevents frameshifts and maintains the anticodon loop structure [93–96].

In summary, tRNA modifications generate diversity and ensure proper folding, especially of the anticodon loop, critical for translation efficiency and translocation [97].

#### 3.2.5. The emerging relevance of epitranscriptomics

Initially a niche field due to the limited number of described modified residues and a lack of understanding of their cellular roles, RNA modification research has advanced significantly with recent technological breakthroughs. While systematic studies of modification diversity and dynamics have been conducted in only a few species, next-generation sequencing (NGS), large-scale epitranscriptome mapping, and the identification of new RNA-modifying enzymes via reverse genetics and mass spectrometry have revolutionized the field [98,99].

The conservation and remarkable diversity of RNA-modifying genes across species, which exceed the essential requirements for canonical base synthesis as mentioned above, highlight their crucial role in biological processes.

### 3.3. tRNA modifications for bacterial phenotypic adaptation

#### 3.3.1. The concept of modification tunable transcripts

Translation efficiency depends on the balance between tRNA supply and the cell’s translational demand [100]. When aminoacyl-tRNAs meet demand, translation proceeds efficiently and accurately. Under stress, rapid synthesis of key proteins is crucial for adaptation. One proposed model suggests that bacteria reprogramme tRNA modifications to modulate translation, adjusting tRNA abundance and codon decoding to selectively express stress response proteins [57,101,102].

These proteins are encoded by modification tunable transcripts (MoTTs), transcripts enriched in codons decoded by dynamically modified tRNAs. Endres *et al*. [101] defined MoTTs as transcripts that (i) encode stress-response proteins, (ii) exhibit specific codon biases and (iii) are translationally regulated by wobble base modifications. MoTTs often contain ‘rare’ codons, typically associated with low-abundance tRNAs that increase under stress, enabling selective translation.

In fact, codon usage, though influenced by species GC content, frequently clusters in genes with related functions [103]. Codon patterns vary between genes and may include enrichment of specific codons [104], codon stretches [94,105] or codon pair/triplet combinations [106]. Over evolutionary time, codon usage and tRNA modification profiles may co-adapt to optimize translation efficiency and conserve energy during protein synthesis [107].

The role of tRNA modifications in codon decoding has been demonstrated across various modifications, codons and organisms. This post-transcriptional layer of regulation adds complexity beyond classical transcriptional control.

#### 3.3.2. Modification tunable transcripts and adaptation to antibiotics and fluctuating oxygen concentrations

To survive antibiotics, bacteria have developed diverse strategies [108]. While resistance, growth at high antibiotic concentrations, is generally well characterized, treatment failure also stems from non-resistant phenotypes like *persistence* and *tolerance* [109]. Tolerant cells, although susceptible, die more slowly due to reduced drug uptake, target activity or stress-induced metabolic slowdown [110]. Unlike certain rRNA modifications, which are directly linked to resistance [111], tRNA modifications are increasingly associated with tolerance.

In *V. cholerae*, high-throughput screens identified RNA modification genes involved in responses to various antibiotics [112], though mechanisms, MoTT-related or not, remain unclear. One key step in tolerance is antibiotic uptake, reliant on proper expression of outer membrane (OM) proteins. In *E. coli* and *Salmonella*, OM proteins like LolB, OmpA and TolC depend on TrmD-mediated m¹G37 tRNA methylation, which enhances translation of proline-rich codons under stress [113]. TrmD, essential in bacteria, regulates m¹G37 levels affecting tRNAs for Pro, Leu and Arg; loss of this modification leads to uncharged tRNA accumulation and ribosome stalling [114], especially in Pro-rich cold-shock transcripts. Similarly, TusB-dependent tRNA modification promotes doxycycline tolerance in *Yersinia pseudotuberculosis* [115].

As previously noted, oxidative and antibiotic stresses are closely linked. In *V. cholerae*, Tgt-mediated queuosine (Q) modification of tRNA^Tyr^ is critical for aminoglycoside tolerance [87,112]. Induced by sub-MIC tobramycin, Tgt enhances oxidative stress responses by modulating SoxR regulon expression via codon-dependent translation of its repressor, the Rsx complex [87]. Bioinformatics also links Tgt to MoTT-regulated genes involved in biofilm formation in *E. coli* [103] and metal homeostasis [116].

In *Pseudomonas aeruginosa*, oxidative stress induces TrmB, increasing m⁷G modification on tRNA^Phe^ and tRNA^Asp^. This boosts translation of catalase genes (*katA*, *katB*) enriched in phenylalanine and aspartic acid codons, enhancing oxidative stress resistance [104]. The MoTT feedback loop is clear: stress → TrmB induction → m⁷G increase tRNAs Asp and Phe → selective translation of catalase transcripts → restored homeostasis. Similar codon enrichment is observed in *E. coli* catalases (*katE*, *katG*), and ∆*trmB* strain show growth defects under peroxide stress [117]. The exact mechanism by which position 46 m⁷G affects decoding remains unclear.

In colistin-resistant *Acinetobacter baumannii*, ∆*trmB* mutants lacking m⁷G on specific tRNAs show heightened oxidative stress sensitivity, reduced macrophage replication and lower infectivity [118]. This is partly due to failure to induce siderophore biosynthesis and uptake proteins, though the codon bias underlying this effect remains untested. *TrmB* is also induced by tigecycline [119], alongside an uncharacterized tRNA methyltransferase [120], though neither has been directly linked to tigecycline resistance [118].

In *M. bovis* BCG, a complete MoTT loop has been described under hypoxia: CmoM-mediated cmo⁵U modification of tRNA^Thr^ enhances translation of ACG-enriched transcripts, including DosR, a master regulator of hypoxic persistence [59].

The general stress sigma factor RpoS is a confirmed MoTT in *E. coli*, helping to limit ROS accumulation during antibiotic stress [35]. Its stability depends on IraP in *E. coli* [121], and both *rpoS* and *iraP* require efficient decoding of leucine codons mediated by the MiaA-catalysed *ms²i⁶A³⁷* tRNA modification [96,122], along with contributions from TrmL and TusA [123]. Though the specific stress signals enhancing leucine codon decoding remain unknown, *miaA* itself is post-transcriptionally regulated by small RNAs [124]. MiaA also affects codon decoding in other contexts, including the RNA chaperone Hfq in *E. coli* ^241^ and secondary metabolism genes in *S. albus*, a producer of bioactive compounds [125].

Stress resistance is tightly linked to host colonization. Oxidative stress-sensitive bacteria, for instance, show reduced infectivity due to macrophage-induced ROS. In *M. tuberculosis*, MnmA-mediated uridine sulfuration supports intracellular growth, likely by enhancing oxidative stress responses [126], also observed in *E. coli* [127]. Like RpoS [122], MiaA-dependent *ms²i⁶A³⁷* and Tgt-mediated *Q* modifications are required for translating leucine-rich virulence genes such as *virF* in *Shigella flexneri* [128]. Similarly, mnm⁵s²U³⁴ synthesis by GidA-MnmE supports virulence in *E. coli* and *Salmonella* [129], while GidA affects *P. aeruginosa* pathogenicity by modulating decoding of rare codons (e.g. AGA, GGA, UUA [130]). Some tRNA modifications even influence host responses, e.g. stress-regulated TrmH-mediated 2′-O-methylation of tRNA^Tyr^ in *E. coli* may help evade immune defences [131,132].

While most examples above involve stress-induced upregulation of RNA modification enzymes, some act as direct sensors. In *E. coli*, MiaB, an iron–sulfur enzyme, modifies codons in the *uof-fur* operon and is activated by iron, allowing post-transcriptional regulation of the iron stress response [133]. Similarly, in *Enterococcus faecalis*, RlmN, a dual tRNA/rRNA methyltransferase, senses ROS via inactivation of its iron–sulphur cluster, triggering superoxide dismutase expression and reducing virulence [134]. In *Escherichia coli*, DusA/B/C also respond to oxidative stress by sensing NADPH levels, adjusting dihydrouridine modification activity accordingly [135]. These enzymes illustrate how RNA modification machinery can act as metabolic or redox sensors, linking environmental cues to translational control.

Though codon dependencies remain poorly characterized, several studies link specific tRNA modifications to the expression of stress response genes. In *S. typhimurium*, deletion of GidA, which installs the mnm⁵s²U³⁴ modification, leads to downregulation of oxidative stress proteins like YghA and Tpx [136]. In *P. aeruginosa*, TrmJ-mediated 2′-O-methylation at position 32 (when A, C or U) is essential for OxyR expression and its regulon [137]. In uropathogenic *E. coli*, TusBCD/MnmA-driven tRNA sulfuration is required for synthesizing virulence factors such as flagella and fimbriae [138]. Loss of tRNA modification enzymes frequently affects motility-related traits [139], underscoring their role in virulence and stress adaptation.

#### 3.3.3. Non-modification tunable transcripts responses and general antibiotic phenotypes

**Figure 4.**
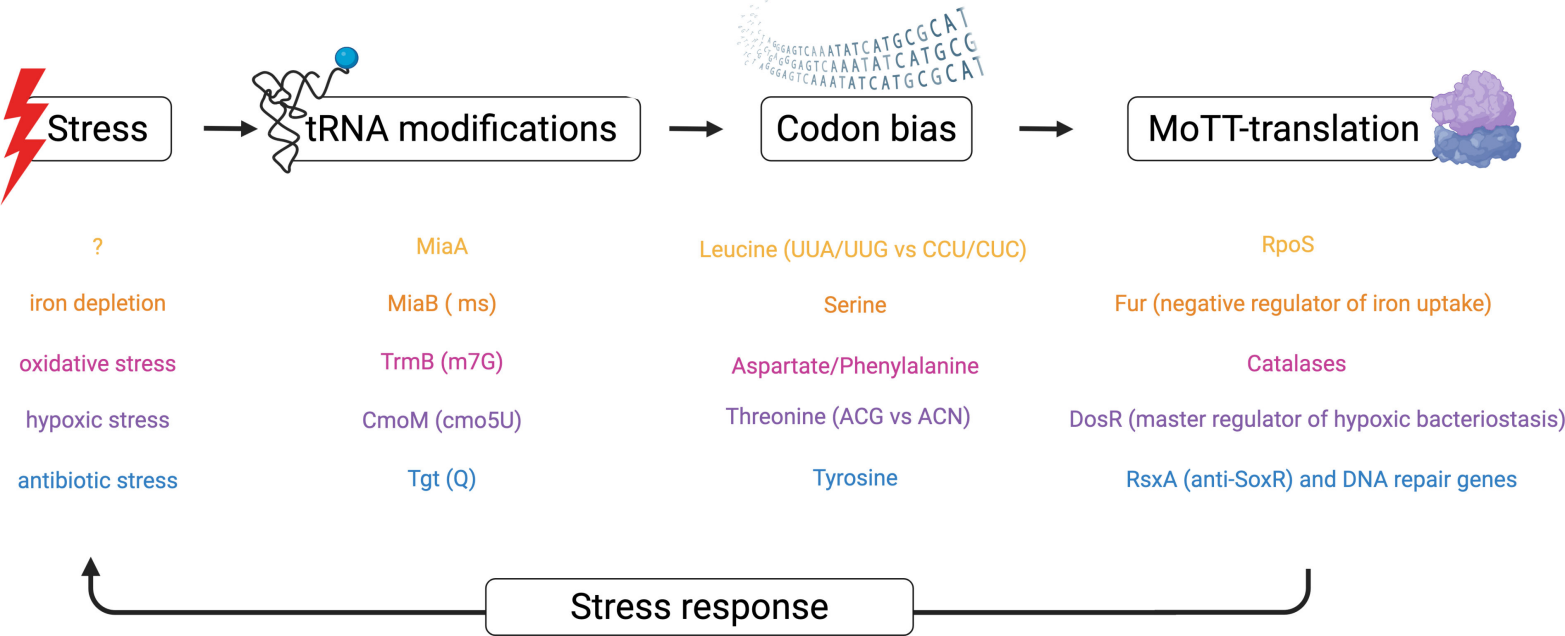
Modulation of bacterial translation under stress through tRNA modification and codon bias. Environmental and intracellular stresses can induce specific tRNA-modifying enzymes, altering tRNA modifications profiles. These epitranscriptomics changes modulate the decoding capacity and structural properties of tRNAs, influencing translation of mRNAs enriched in specific codons, referred to as modification tunable transcriptome (MoTT). This mechanism enables the selective synthesis of stress-responsive proteins, contributing to bacterial adaptation and survival under adverse conditions.

Beyond MoTT-based mechanisms (figure 4), stress adaptation can also result from tRNA structural changes mediated by modifications. In thermophilic archaea, such modifications stabilize tRNA against heat-induced destabilization, preserving translation efficiency [60]. Similar roles are likely in other organisms' heat shock responses [61].

Under oxidative stress in *E. coli*, Tgt has been shown to damage its substrate tRNAs by introducing abasic sites at the anticodon, potentially reducing translation during stress [140]. Additionally, a recent study identified 5-methylcytidine (m⁵C) at position 49 of *E. coli* tRNA^Tyr^, catalysed by RsmF, an rRNA methyltransferase induced under oxidative and heat stress [141]. Although ∆*rsmF* mutants are ROS-sensitive, the precise role of m⁵C in stress resilience remains unclear. This finding is particularly interesting because it was previously thought that m5C was not found on bacterial tRNAs.

UV irradiation, another oxidative stressor, also alters RNA modification profiles [142]. In *E. coli*, UV-induced crosslinking of 4-thiouridine at position 8 with cytidine at position 13 in tRNA^Phe^ and tRNA^Pro^ disrupts aminoacylation, halts translation, and temporarily inhibits growth [143], likely a reversible protective mechanism against UV damage.

On a different note, characterizing redox proteins, such as metal cofactor enzymes and flavoproteins, can shed light on their roles in managing or exacerbating oxidative stress. Notably, several tRNA-modifying enzymes, like the flavoprotein dihydrouridine synthases DusA, DusB and DusC, participate in redox reactions [144,145] and modulate their activity in response to the intracellular redox state [135]. Under oxidative stress, their reduced activity lowers dihydrouridine (D) levels on tRNAs, raising the question of whether D contributes to stress adaptation.

tRNA-modifying genes impact translation, regulation and stress responses, making them potential targets to combat antibiotic resistance [3] and bacterial virulence [130,146]. As proof-of-concept, the efficacy of fluoropyrimidines like 5-fluorouracil (5-FU) and 5-fluorocytosine (5-FC) partly stems from their disruption of U-modifications (e.g. m⁵U and Ψ) in tRNAs, leading to tRNA destabilization [147]. Pre-existing tRNA hypomodification amplifies these effects, suggesting RNA-modifying enzymes could be leveraged to boost the potency of such treatments.

In some cases, tRNAs undergo post-transcriptional cleavage to generate tRNA-derived fragments (tRFs), including halves and shorter pieces (13–20 nt) [148]. First identified in human tumours [149] and phage-infected *E. coli* [150], tRF formation is conserved across all domains of life [151]. In bacteria, tRFs regulate diverse processes, such as gene expression [152] and host cell survival in *M. tuberculosis* [153]. While direct links between tRFs and RNA modifications are well-established in eukaryotes [154–156], this connection remains to be fully explored in bacteria.

tRNAs also participate in non-translational functions [37,148,157]. In *Streptomyces*, tRNA-delivered amino acids serve as precursors for antibiotic biosynthesis [158]. tRNAs can also donate amino acids for peptidoglycan synthesis [159], membrane lipid aminoacylation and N-terminal protein tagging, affecting protein stability and degradation [160].

Given the broad roles of tRNA modifications, their loss often causes pleiotropic effects, making it difficult to separate the impact of the modification from that of the enzyme. For instance, while *E. coli* pseudouridine synthases (e.g. *pus* genes) are dispensable under optimal growth, a ∆*truB* mutant is outcompeted by wild-type at 37°C, independently of TruB’s enzymatic activity [161]. This introduces the next topic: the roles of tRNA-modifying enzymes beyond their catalytic functions.

## 4. Beyond modification: other roles for tRNA-modifying enzymes

This section highlights that phenotypic effects may stem from either the modifications or the enzymes responsible for catalysing them, which can have distinct roles.

### 4.1. Dual function tRNA modification enzymes

Some tRNA-modifying enzymes have dual functions, either modifying multiple RNA species or performing additional, non-catalytic roles. For instance, RlmN [162], RluF [163] and RluA [164] modify both tRNAs and rRNAs. Notably, RluA was recently shown to pseudouridylate bacterial mRNAs [165], specifically targeting structured motifs in hairpin loops.

Beyond catalytic duality, several enzymes moonlight in non-modification roles. MiaB, for example, influences type III secretion in *P. aeruginosa* independently of its role in tRNA thiolation. This regulatory function may involve direct interaction with transcription factors, as it co-purifies with the global regulator Vfr [166]. Further studies are needed to elucidate the underlying mechanism.

Another example comes from a recent study showing that antimicrobial peptides increase the expression of QueE in *E. coli*. QueE is a key enzyme in queuosine biosynthesis for tRNAs (Asp, Asn, His, Tyr), and was recently shown to block cell division at the septal ring in response to antimicrobial peptides, a role unrelated to tRNA modification [167]. This moonlighting function appears conserved in *E. coli*, *S. typhimurium* and *K. pneumoniae*, but not in *P. aeruginosa* or *B. subtilis*, suggesting species-specific adaptation.

Another striking case is TilS, essential for modifying tRNAᶦˡᵉ to ensure translational fidelity. In *Burkholderia cenocepacia* and *E. coli*, mutations in *tilS* that disrupt its catalytic function confer growth advantages under redox-imbalanced conditions. This suggests a trade-off between translation accuracy (via modified tRNAᶦˡᵉ) and metabolic fitness under redox-imbalanced conditions, via catalytically inactive TilS, pointing to a secondary role of TilS in redox homoeostasis [168].

These examples highlight that tRNA-modifying enzymes are not only central to translation, but also contribute to broader cellular functions such as stress response, cell division and virulence, often through mechanisms beyond their canonical enzymatic activity.

### 4.2. tRNA modifying enzymes as tRNA chaperones

tRNA structure and stability are essential for proper function. For example, efficient aminoacylation depends on correct folding. While tRNA processing is generally well-coordinated [169], proper folding into the native L-shaped three-dimensional structure is a critical maturation step, since aberrant, non-functional conformations can be energetically favourable and rapidly adopted [170,171]. Misfolding arises primarily from: (i) incorrect base pairing that is hard to reverse, and (ii) difficulties in achieving productive folding over energetically stable misfolded states. As a result, RNAs, including tRNAs, often become kinetically trapped in non-productive conformations [172,173], and only a minority reach their active state [174]. This contributes to longer folding timescales compared to proteins [175].

**Figure 5.**
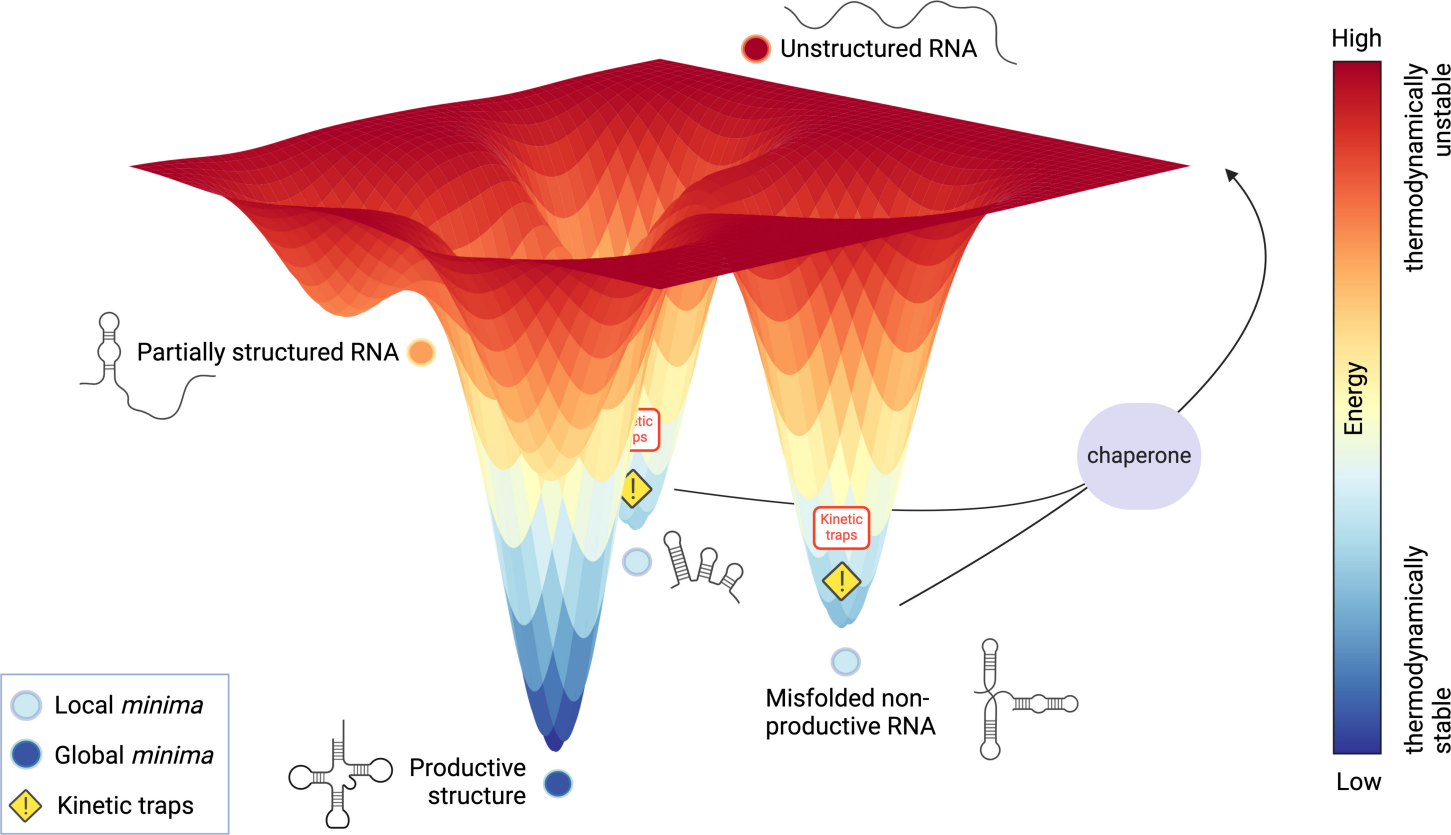
RNA folding energy landscape and kinetic traps (adapted from [177], generated using ChatGPT). This schematic represents a theoretical three-dimensional RNA folding energy landscape, where colour gradients correspond to energy levels (red: high energy; dark blue: low energy). Unfolded RNA is associated with high-energy (red zone), while folding proceeds downhill towards energy minima. Light blue regions represent *local minima* associated with incorrect pairings and non-productive conformations, also named *kinetic traps*. In contrast, the global minima, represented in dark blue, corresponds to the native, productive structure. RNA chaperones facilitate correct folding by helping misfolded RNAs to escape from kinetic traps. Theorical RNA structures are depicted along the folding trajectory for illustrative purposes.

To overcome the challenges of RNA misfolding, organisms rely on RNA chaperones, proteins that assist RNA in reaching its correct conformation. They destabilize incorrect base-pairing, helping RNAs escape kinetic traps through a process known as iterative annealing [171,176] (figure 5). Unlike protein chaperones, RNA chaperones act in an energy-independent manner, relying on thermodynamic differences between native and misfolded conformations. To date, very few tRNA chaperones have been identified [178]. The best-characterized is the human La protein, which binds pre-tRNAs with high affinity to aid maturation and protect them from exonucleolytic decay [179]. In bacteria, only two tRNA chaperones are known so far, remarkably, these are the RNA-modifying enzymes TruB and TrmA [53,180,181].

Verifying chaperone activity in tRNA-modifying enzymes is challenging due to functional redundancy, as loss of one enzyme often has minimal impact [180]. Additionally, when tRNA destabilization is observed in knockout strains, it is hard to separate the effects of lost modification from the loss of chaperone activity.

In *E. coli*, ∆*truB* causes moderate growth defects under normal conditions, which become more pronounced under stress [79,161,180]. However, catalytically inactive TruB variants that still bind tRNA can rescue the fitness defects of ∆*truB* strains. TruB acts as a tRNA chaperone by transiently binding both folded and misfolded tRNAs, disrupting tertiary interactions in the elbow region (D- and T-arms), and promoting proper folding for aminoacylation, all independently of ATP or its Ψ55 synthase activity [180]. This chaperone role is essential for fitness under stress, as shown in co-culture assays [161,180]. Nonetheless, Ψ55 itself also aids survival during heat and cold shock [79,80].

Similarly, TrmA, a SAM-dependent methyltransferase that installs m⁵U at position 54, shows binding-dependent, catalysis-independent chaperone activity *in vitro* [181]. Like TruB, it targets the tRNA elbow, locally unfolding the T-loop to access its substrate [182]. However, dissecting TrmA’s chaperone role is harder since m⁵U is essential for growth. Recent studies using catalytic mutants show that m⁵U54 contributes to resistance against translation elongation inhibitors like hygromycin B and ensures full modification under stress [183].

Might all RNA-modifiers moonlight as chaperones? Although RNA chaperones are often considered non-specific RNA binders [184], tRNA chaperones stand out by their selectivity: they may bind all tRNAs but do not interact with other RNA types. This mirrors the specificity of protein chaperones, which typically assist certain protein classes rather than all peptide chains [185]. Given that many tRNA-modifying enzymes promote structural rearrangements [182,186–188], including local unwinding or base-flipping, to access their target sites, their chaperone-like activity seems intuitive. The Kothe lab proposed that such remodelling of tRNA structure, particularly in the elbow region critical for three-dimensional folding [53,180], may underpin the chaperone functions of modification enzymes [53]. This highlights the close link between tRNA folding, maturation and modification. A similar phenomenon is observed in ribosomal RNA, where certain modifying enzymes, like the methyltransferase RsmC, also exhibit chaperone activity [189]*.*

## 5. Future directions

### 5.1. Expanding knowledge of tRNA modifications across species and environments

To date, the complete landscape of tRNA modifications and their corresponding enzymes has been characterized in only a few bacterial species, including *E. coli* [190], *Mycoplasma capricolum* [191] and *Bacillus subtilis* [192]. More recently, in 2024, comprehensive tRNA modification profiles have also been mapped in *Pseudomonas aeruginosa* [193] and *Bartonella* [194]. Partial modification maps also exist for *Staphylococcus aureus [195]*, *Methanocaldococcus jannaschii* [196], *Streptomyces albus* [197] and thermophilic aerobic bacilli [198], although the corresponding enzymes remain unidentified in many cases.

New tRNA modifications and enzymes continue to be discovered, such as aminovaleramide [199], *V. cholerae*-specific cytidine-to-pseudouridine editing [200], a flavin-dependent methyltransferase [201] and several others [202–204]. High-throughput techniques are increasingly used to study regulatory networks governing tRNA modifications [205–207], including responses to heat stress [208], temperature shifts [207,209], nutrient availability [210,211], phage infection [62,212] and phage-encoded tRNAs [213].

Transcriptomics and genomic studies across various environments and species have been conducted over many years, providing valuable data on the expression and fitness of different tRNA modification genes. A differential gene expression profile between *in vivo* (patient) and *in vitro* (urine) conditions in uropathogenic *E. coli* (UPEC), revealed that genes involved in tRNA modification, including *queA*, *thiI*, miaB, tsaB, *dusC, tadA, truC, tcdA, dusB, tusA* and *trmA*, were among those significantly upregulated during urinary tract infection (UTI) [214]. Supporting this, MiaA was also found to be required for maximal fitness in mouse UTI and systemic infection models of extraintestinal pathogenic *E. coli* (ExPEC) [215]. Shea *et al.* further identified the tRNA modification genes *thdF* and *gidA* as important fitness factors for *E. coli* CFT073 during UTI, necessary for bacterial growth in human urine and effective urinary tract colonization [216]. Similarly, deletion of the *tusDCB* sulfur relay system in UPEC impaired the production of virulence factors such as type 1 fimbriae and flagella, and reduced bacterial aggregation in bladder epithelial cells [138]. Beyond *E. coli*, other pathogens also rely on tRNA modifications for successful infection. In *P. aeruginosa*, GidA [130], TrmA [217] and TrhPO [218] were shown to be essential for pathogenicity by facilitating translation of virulence genes involved in motility, cytotoxicity, biofilm formation and overall fitness. In *V. cholerae*, a transposon insertion sequencing screen in neonatal rabbits identified several tRNA modification genes, including *mnmE*, *gidA* and *thiI*, as critical for intestinal colonization [219]. A separate high-coverage Tn-seq analysis in *V. cholerae* E7946 also found *thiI*, *truA*, *queA* and *tgt* to be essential or strongly required for both intestinal colonization and survival in pond water [220]. During *Shigella* infection an organoid model uncovered the role of MnmE and GidA on host colonization [221]. In plant pathogens, a genome-wide screen during *Xanthomonas hortorum pv. vitians* infection in lettuce showed that disruption of *tgt*, severely compromised bacterial fitness in plants [222]. As a result, post-transcriptional tRNA modifications are increasingly recognized for having a role in bacterial fitness and virulence across diverse infection models.

### 5.2. Modification networks

Many unknowns remain about modification networks, including the precise order of modifications and their mutual influence [76]. Few studies have systematically examined combinations of modification deficient mutants [223]. While single gene deletions rarely cause growth defects, combined mutations may reveal compensatory interactions.

### 5.3. New challenges

Despite the constraints, new approaches continue to emerge [224]. For instance, nanopore technology now enables direct analysis of aminoacylation on native tRNAs [225], facilitating studies on how individual modifications affect charging efficiency. The concept of metaepitranscriptomics, the study of tRNA modifications within microbiomes and natural communities, has also been proposed [206], though such studies have yet to be reported and practical applications are still in early stages.

It is important to note that, in addition to tRNA modifications, ribosomal RNA (rRNA) modifications have also been shown to play roles in cellular adaptation to stress. In particular, certain rRNA-modifying enzymes respond dynamically to environmental conditions, contributing to ribosome heterogeneity and functional reprogramming under stress [226–228]. Given the mechanistic parallels between rRNA and tRNA modification systems, including overlapping enzymes and shared regulatory pathways, approaches and technologies developed for the study of rRNA modifications, such as high-throughput sequencing, structure-probing techniques or real-time modification mapping, could be adapted or repurposed to advance our understanding of tRNA modifications. As such, cross-applications of these methodologies may offer promising avenues for future research into the regulatory dynamics of the tRNA epitranscriptome.

## 6. Conclusion

A recent NASEM report highlights the importance of mapping RNA modifications in various species and conditions [229]. In line with this, this review addresses a critical and timely topic, from a bacterial stress-response perspective. While our focus is on tRNA modifications, recent reviews on rRNA modifications also emphasize the role of modification-driven ribosome heterogeneity and dynamics [226,227].

The field of RNA modifications in bacteria has long been underexplored. While tRNA and rRNA modifications have been extensively studied in eukaryotes, often in the context of human disease, their roles in bacterial stress response and adaptation remain poorly characterized. This gap is partly due to the lack of clear phenotypes in unstressed cells when many RNA modification genes are deleted, despite some being essential [230]. Additionally, technical challenges have limited the detection of these modifications.

Interest in bacterial RNA modifications is growing, driven by recent studies linking tRNA modifications to stress responses. Research on SARS-CoV-2 has further boosted attention to RNA modifications, revealing their roles in viral infection and host immunity [231]. Notably, the success of mRNA vaccines against SARS-CoV-2 is partly due to the methyl-pseudouridine (m¹Ψ) modification, which improves translation efficiency [232].

In recent years, Illumina-based chemical detection methods have been developed to identify a broad range of tRNA and rRNA modifications [233–237]. While these techniques have enabled important discoveries using simple RNA extracts, they have notable limitations. Short read lengths make it difficult to study multiple modifications on a single RNA molecule or distinguish closely related RNA species, such as similar rRNA operons. Low-abundance RNAs may also go undetected, and the technical complexity limits their use to a few specialized laboratories. Importantly, newly identified modifications require validation through multiple approaches, particularly when the responsible enzyme is unknown and cannot be disrupted to confirm loss of the modification. At the same time, advances in nanopore sequencing are rapidly improving RNA modification detection, offering promising avenues for future research.

tRNA modifications in bacteria offer an exciting frontier for novel therapeutic strategies [238–241]. Although often non-essential under standard conditions, these modifications play key roles in bacterial physiology and pathogenicity. Targeting tRNA-modifying enzymes or pathways can disrupt processes such as virulence, motility, host colonization and the expression of antibiotic-resistance genes [129,146,242]. Some modifications enhance the translation of resistance factors, stress regulators or efflux pumps, interfering with them could weaken bacterial fitness or increase antibiotic sensitivity without imposing strong selective pressure.

Altogether, the study of tRNA modifications holds great promise, offering new insights into bacterial physiology and potential therapeutic avenues, with many questions still unanswered and exciting discoveries ahead.

## Data Availability

This article has no additional data.

## References

[1] Kimura S, Srisuknimit V, Waldor MK. 2020 Probing the diversity and regulation of tRNA modifications. Curr. Opin. Microbiol. **57**, 41–48. (10.1016/j.mib.2020.06.005) 32663792 PMC7722113

[2] Pollo-Oliveira L, de Crecy-Lagard V. 2019 Can protein expression be regulated by modulation of tRNA modification profiles? Biochemistry **58**, 355–362. (10.1021/acs.biochem.8b01035) 30511849 PMC6363828

[3] de Crecy-Lagard V, Jaroch M. 2021 Functions of bacterial tRNA modifications: from ubiquity to diversity. Trends Microbiol. **29**, 41–53. (10.1016/j.tim.2020.06.010) 32718697 PMC8865055

[4] Barraud P, Tisné C. 2019 To be or not to be modified: miscellaneous aspects influencing nucleotide modifications in tRNAs. IUBMB Life **71**, 1126–1140. (10.1002/iub.2041) 30932315 PMC6850298

[5] Hofer K, Jaschke A. 2018 Epitranscriptomics: RNA modifications in bacteria and archaea. Microbiol. Spectr. **6**. (10.1128/microbiolspec.rwr-0015-2017) PMC11633594 29916347

[6] Edwards AM, Addo MA, Dos Santos PC. 2020 Extracurricular functions of tRNA modifications in microorganisms. Genes **11**, 907. (10.3390/genes11080907) 32784710 PMC7466049

[7] Yared MJ, Marcelot A, Barraud P. 2024 Beyond the anticodon: tRNA core modifications and their impact on structure, translation and stress adaptation. Genes **15**, 374. (10.3390/genes15030374) 38540433 PMC10969862

[8] Lyons TW, Reinhard CT, Planavsky NJ. 2014 The rise of oxygen in Earth’s early ocean and atmosphere. Nature **506**, 307–315. (10.1038/nature13068) 24553238

[9] Di Mascio P, Martinez GR, Miyamoto S, Ronsein GE, Medeiros MHG, Cadet J. 2019 Singlet molecular oxygen reactions with nucleic acids, lipids, and proteins. Chem. Rev. **119**, 2043–2086. (10.1021/acs.chemrev.8b00554) 30721030

[10] Halliwell B, Gutteridge JM. 1984 Oxygen toxicity, oxygen radicals, transition metals and disease. Biochem. J. **219**, 1–14. (10.1042/bj2190001) 6326753 PMC1153442

[11] Lushchak VI. 2011 Adaptive response to oxidative stress: bacteria, fungi, plants and animals. Comp. Biochem. Physiol. C Toxicol. Pharmacol. **153**, 175–190. (10.1016/j.cbpc.2010.10.004) 20959147

[12] Sies H. 1997 Oxidative stress: oxidants and antioxidants. Exp. Physiol. **82**, 291–295. (10.1113/expphysiol.1997.sp004024) 9129943

[13] Seaver LC, Imlay JA. 2001 Hydrogen peroxide fluxes and compartmentalization inside growing Escherichia coli. J. Bacteriol. **183**, 7182–7189. (10.1128/jb.183.24.7182-7189.2001) 11717277 PMC95567

[14] Hopwood MJ, Rapp I, Schlosser C, Achterberg EP. 2017 Hydrogen peroxide in deep waters from the Mediterranean Sea, South Atlantic and South Pacific Oceans. Sci. Rep. **7**, 43436. (10.1038/srep43436) 28266529 PMC5339902

[15] Cooper WJ, Zika RG, Petasne RG, Plane JM. 1988 Photochemical formation of hydrogen peroxide in natural waters exposed to sunlight. Environ. Sci. Technol. **22**, 1156–1160. (10.1021/es00175a004) 22148607

[16] Dahlgren C, Karlsson A. 1999 Respiratory burst in human neutrophils. J. Immunol. Methods **232**, 3–14. (10.1016/s0022-1759(99)00146-5) 10618505

[17] Andres C, Perez de la Lastra JM, Juan CA, Plou FJ, Perez-Lebena E. 2022 The role of reactive species on innate immunity. Vaccines **10**, 1735. (10.3390/vaccines10101735) 36298601 PMC9609844

[18] Qadri F, Raqib R, Ahmed F, Rahman T, Wenneras C, Das SK, Alam NH, Mathan MM, Svennerholm AM. 2002 Increased levels of inflammatory mediators in children and adults infected with Vibrio cholerae O1 and O139. Clin. Diagn. Lab. Immunol. **9**, 221–229. (10.1128/cdli.9.2.221-229.2002) 11874856 PMC119937

[19] Ellis CN *et al*. 2015 Comparative proteomic analysis reveals activation of mucosal innate immune signaling pathways during cholera. Infect. Immun. **83**, 1089–1103. (10.1128/IAI.02765-14) 25561705 PMC4333457

[20] Jakubovics NS, Gill SR, Vickerman MM, Kolenbrander PE. 2008 Role of hydrogen peroxide in competition and cooperation between Streptococcus gordonii and Actinomyces naeslundii. FEMS Microbiol. Ecol. **66**, 637–644. (10.1111/j.1574-6941.2008.00585.x) 18785881 PMC2820160

[21] Manyi-Loh C, Mamphweli S, Meyer E, Okoh A. 2018 Antibiotic use in agriculture and its consequential resistance in environmental sources: potential public health implications. Molecules **23**, 795. (10.3390/molecules23040795) 29601469 PMC6017557

[22] Casewell M, Friis C, Marco E, McMullin P, Phillips I. 2003 The European ban on growth-promoting antibiotics and emerging consequences for human and animal health. J. Antimicrob. Chemother. **52**, 159–161. (10.1093/jac/dkg313) 12837737

[23] Haggard BE, Bartsch LD. 2009 Net changes in antibiotic concentrations downstream from an effluent discharge. J. Environ. Qual. **38**, 343–352. (10.2134/jeq2007.0540) 19141825

[24] Chow LKM, Ghaly TM, Gillings MR. 2021 A survey of sub-inhibitory concentrations of antibiotics in the environment. J. Environ. Sci. **99**, 21–27. (10.1016/j.jes.2020.05.030) 33183698

[25] Fick J, Söderström H, Lindberg RH, Phan C, Tysklind M, Larsson DG. 2009 Contamination of surface, ground, and drinking water from pharmaceutical production. Environ. Toxicol. Chem. **28**, 2522–2527. (10.1897/09-073.1) 19449981

[26] Davies J, Spiegelman GB, Yim G. 2006 The world of subinhibitory antibiotic concentrations. Curr. Opin. Microbiol. **9**, 445–453. (10.1016/j.mib.2006.08.006) 16942902

[27] Hughes D, Andersson DI. 2012 Selection of resistance at lethal and non-lethal antibiotic concentrations. Curr. Opin. Microbiol. **15**, 555–560. (10.1016/j.mib.2012.07.005) 22878455

[28] Andersson DI, Hughes D. 2014 Microbiological effects of sublethal levels of antibiotics. Nat. Rev. Microbiol. **12**, 465–478. (10.1038/nrmicro3270) 24861036

[29] Goh EB, Yim G, Tsui W, McClure J, Surette MG, Davies J. 2002 Transcriptional modulation of bacterial gene expression by subinhibitory concentrations of antibiotics. Proc. Natl Acad. Sci. USA **99**, 17025–17030. (10.1073/pnas.252607699) 12482953 PMC139263

[30] Fajardo A, Martínez JL. 2008 Antibiotics as signals that trigger specific bacterial responses. Curr. Opin. Microbiol. **11**, 161–167. (10.1016/j.mib.2008.02.006) 18373943

[31] Baharoglu Z, Garriss G, Mazel D. 2013 Multiple pathways of genome plasticity leading to development of antibiotic resistance. Antibiotics **2**, 288–315. (10.3390/antibiotics2020288) 27029305 PMC4790341

[32] Brand C, Newton-Foot M, Grobbelaar M, Whitelaw A. 2025 Antibiotic-induced stress responses in Gram-negative bacteria and their role in antibiotic resistance. J. Antimicrob. Chemother. **80**, 1165–1184. (10.1093/jac/dkaf068) 40053699 PMC12046405

[33] Carvalho A, Krin E, Korlowski C, Mazel D, Baharoglu Z. 2021 Interplay between sublethal aminoglycosides and quorum sensing: consequences on survival in V. cholerae. Cells **10**, 3227. (10.3390/cells10113227) 34831448 PMC8621022

[34] Calabrese EJ, Baldwin LA. 2002 Defining hormesis. Hum. Exp. Toxicol. **21**, 91–97. (10.1191/0960327102ht217oa) 12102503

[35] Baharoglu Z, Krin E, Mazel D. 2013 RpoS plays a central role in the SOS induction by sub-lethal aminoglycoside concentrations in Vibrio cholerae. PLoS Genet. **9**, e1003421. (10.1371/journal.pgen.1003421) 23613664 PMC3623755

[36] Kohanski MA, Dwyer DJ, Collins JJ. 2010 How antibiotics kill bacteria: from targets to networks. Nat. Rev. Microbiol. **8**, 423–435. (10.1038/nrmicro2333) 20440275 PMC2896384

[37] Shepherd J, Ibba M. 2015 Bacterial transfer RNAs. FEMS Microbiol. Rev. **39**, 280–300. (10.1093/femsre/fuv004) 25796611 PMC4542688

[38] Cappannini A *et al*. 2024 MODOMICS: a database of RNA modifications and related information. 2023 update. Nucleic Acids Res. **52**, D239–D244. (10.1093/nar/gkad1083) 38015436 PMC10767930

[39] Mohanty BK, Agrawal A, Kushner SR. 2020 Generation of pre-tRNAs from polycistronic operons is the essential function of RNase P in Escherichia coli. Nucleic Acids Res. **48**, 2564–2578. (10.1093/nar/gkz1188) 31993626 PMC7049720

[40] Grosjean H. 2009 Nucleic acids are not boring long polymers of only four types of nucleotides: a guided tour. In Madame Curie Bioscience Database, pp. 2000–2013. Austin, TX: Landes Bioscience. See https://www.ncbi.nlm.nih.gov/books/NBK6489/.

[41] Han L, Phizicky EM. 2018 A rationale for tRNA modification circuits in the anticodon loop. RNA **24**, 1277–1284. (10.1261/rna.067736.118) 30026310 PMC6140457

[42] Motorin Y, Helm M. 2010 tRNA stabilization by modified nucleotides. Biochemistry **49**, 4934–4944. (10.1021/bi100408z) 20459084

[43] Kimura S, Waldor MK. 2019 The RNA degradosome promotes tRNA quality control through clearance of hypomodified tRNA. Proc. Natl Acad. Sci. USA **116**, 1394–1403. (10.1073/pnas.1814130116) 30622183 PMC6347707

[44] Lorenz C, Lünse CE, Mörl M. 2017 tRNA modifications: impact on structure and thermal adaptation. Biomolecules **7**, 35. (10.3390/biom7020035) 28375166 PMC5485724

[45] Clifton BE, Fariz MA, Uechi GI, Laurino P. 2021 Evolutionary repair reveals an unexpected role of the tRNA modification m1G37 in aminoacylation. Nucleic Acids Res. **49**, 12467–12485. (10.1093/nar/gkab1067) 34761260 PMC8643618

[46] Sylvers LA, Rogers KC, Shimizu M, Ohtsuka E, Soll D. 1993 A 2-thiouridine derivative in tRNAGlu is a positive determinant for aminoacylation by Escherichia coli glutamyl-tRNA synthetase. Biochemistry **32**, 3836–3841. (10.1021/bi00066a002) 8385989

[47] Masuda I, McGuigan H, Maharjan S, Yamaki Y, Hou YM. 2025 Connecting tRNA charging and decoding through the axis of nucleotide modifications at position 37. J. Mol. Biol. **437**, 169095. (10.1016/j.jmb.2025.169095) 40113011 PMC12162217

[48] Muramatsu T, Nishikawa K, Nemoto F, Kuchino Y, Nishimura S, Miyazawa T, Yokoyama S. 1988 Codon and amino-acid specificities of a transfer RNA are both converted by a single post-transcriptional modification. Nature **336**, 179–181. (10.1038/336179a0) 3054566

[49] Agris PF. 2004 Decoding the genome: a modified view. Nucleic Acids Res. **32**, 223–238. (10.1093/nar/gkh185) 14715921 PMC384350

[50] Urbonavicius J, Qian Q, Durand JM, Hagervall TG, Bjork GR. 2001 Improvement of reading frame maintenance is a common function for several tRNA modifications. EMBO J. **20**, 4863–4873. (10.1093/emboj/20.17.4863) 11532950 PMC125605

[51] Chanfreau GF. 2017 Impact of RNA modifications and RNA-modifying enzymes on eukaryotic ribonucleases. Enzymes **41**, 299–329. (10.1016/bs.enz.2017.03.008) 28601225

[52] Cochella L, Green R. 2005 An active role for tRNA in decoding beyond codon:anticodon pairing. Science **308**, 1178–1180. (10.1126/science.1111408) 15905403 PMC1687177

[53] Schultz SK, Katanski CD, Halucha M, Peña N, Fahlman RP, Pan T, Kothe U. 2024 Modifications in the T arm of tRNA globally determine tRNA maturation, function, and cellular fitness. Proc. Natl Acad. Sci. USA **121**, e2401154121. (10.1073/pnas.2401154121) 38889150 PMC11214086

[54] Sakai Y, Miyauchi K, Kimura S, Suzuki T. 2016 Biogenesis and growth phase-dependent alteration of 5-methoxycarbonylmethoxyuridine in tRNA anticodons. Nucleic Acids Res. **44**, 509–523. (10.1093/nar/gkv1470) 26681692 PMC4737166

[55] Emilsson V, Näslund AK, Kurland CG. 1992 Thiolation of transfer RNA in Escherichia coli varies with growth rate. Nucleic Acids Res. **20**, 4499–4505. (10.1093/nar/20.17.4499) 1383926 PMC334177

[56] Chan CT, Dyavaiah M, DeMott MS, Taghizadeh K, Dedon PC, Begley TJ. 2010 A quantitative systems approach reveals dynamic control of tRNA modifications during cellular stress. PLoS Genet. **6**, e1001247. (10.1371/journal.pgen.1001247) 21187895 PMC3002981

[57] Gu C, Begley TJ, Dedon PC. 2014 tRNA modifications regulate translation during cellular stress. FEBS Lett. **588**, 4287–4296. (10.1016/j.febslet.2014.09.038) 25304425 PMC4403629

[58] Gupta R, Laxman S. 2020 tRNA wobble-uridine modifications as amino acid sensors and regulators of cellular metabolic state. Curr. Genet. **66**, 475–480. (10.1007/s00294-019-01045-y) 31758251

[59] Chionh YH *et al*. 2016 tRNA-mediated codon-biased translation in mycobacterial hypoxic persistence. Nat. Commun. **7**, 13302. (10.1038/ncomms13302) 27834374 PMC5114619

[60] Kowalak JA, Dalluge JJ, McCloskey JA, Stetter KO. 1994 The role of posttranscriptional modification in stabilization of transfer RNA from hyperthermophiles. Biochemistry **33**, 7869–7876. (10.1021/bi00191a014) 7516708

[61] Ohira T, Suzuki T. 2024 Transfer RNA modifications and cellular thermotolerance. Mol. Cell **84**, 94–106. (10.1016/j.molcel.2023.11.041) 38181765

[62] Lampi M, Gregorova P, Qasim MS, Ahlblad NCV, Sarin LP. 2023 Bacteriophage infection of the marine bacterium Shewanella glacialimarina induces dynamic changes in tRNA modifications. Microorganisms **11**, 355. (10.3390/microorganisms11020355) 36838320 PMC9963407

[63] Begik O *et al*. 2021 Quantitative profiling of pseudouridylation dynamics in native RNAs with nanopore sequencing. Nat. Biotechnol. **39**, 1278–1291. (10.1038/s41587-021-00915-6) 33986546

[64] Furlan M, Delgado-Tejedor A, Mulroney L, Pelizzola M, Novoa EM, Leonardi T. 2021 Computational methods for RNA modification detection from nanopore direct RNA sequencing data. RNA Biol. **18**, 31–40. (10.1080/15476286.2021.1978215) 34559589 PMC8677041

[65] Lucas MC, Pryszcz LP, Medina R, Milenkovic I, Camacho N, Marchand V, Motorin Y, Ribas de Pouplana L, Novoa EM. 2024 Quantitative analysis of tRNA abundance and modifications by nanopore RNA sequencing. Nat. Biotechnol. **42**, 72–86. (10.1038/s41587-023-01743-6) 37024678 PMC10791586

[66] Jamontas R, Laurynenas A, Povilaityte D, Meskys R, Aucynaite A. 2024 RudS: bacterial desulfidase responsible for tRNA 4-thiouridine de-modification. Nucleic Acids Res. **52**, 10543–10562. (10.1093/nar/gkae716) 39166491 PMC11417400

[67] Foo M, Frietze LR, Enghiad B, Yuan Y, Katanski CD, Zhao H, Pan T. 2024 Prokaryotic RNA N1-methyladenosine erasers maintain tRNA m1A modification levels in Streptomyces venezuelae. ACS Chem. Biol. **19**, 1616–1625. (10.1021/acschembio.4c00278) 38912606

[68] Shi H, Wei J, He C. 2019 Where, when, and how: context-dependent functions of RNA methylation writers, readers, and erasers. Mol. Cell **74**, 640–650. (10.1016/j.molcel.2019.04.025) 31100245 PMC6527355

[69] Prossliner T, Agrawal S, Heidemann DF, Sorensen MA, Svenningsen SL. 2023 tRNAs are stable after all: pitfalls in quantification of tRNA from starved Escherichia coli cultures exposed by validation of RNA purification methods. mBio **14**, e0280522. (10.1128/mbio.02805-22) 36598190 PMC9973347

[70] Chen Z *et al*. 2019 Transfer RNA demethylase ALKBH3 promotes cancer progression via induction of tRNA-derived small RNAs. Nucleic Acids Res. **47**, 2533–2545. (10.1093/nar/gky1250) 30541109 PMC6411830

[71] Jia G *et al*. 2011 N6-Methyladenosine in nuclear RNA is a major substrate of the obesity-associated FTO. Nat. Chem. Biol. **7**, 885–887. (10.1038/nchembio.687) 22002720 PMC3218240

[72] Liu F *et al*. 2016 ALKBH1-mediated tRNA demethylation regulates translation. Cell **167**, 816–828.(10.1016/j.cell.2016.09.038) 27745969 PMC5119773

[73] Ueda Y *et al*. 2017 AlkB homolog 3-mediated tRNA demethylation promotes protein synthesis in cancer cells. Sci. Rep. **7**, 42271. (10.1038/srep42271) 28205560 PMC5304225

[74] Wei J *et al*. 2018 Differential m(6)A, m(6)A(m), and m(1)A demethylation mediated by fto in the cell nucleus and cytoplasm. Mol. Cell **71**, 973–985. (10.1016/j.molcel.2018.08.011) 30197295 PMC6151148

[75] Zheng G *et al*. 2013 ALKBH5 is a mammalian RNA demethylase that impacts RNA metabolism and mouse fertility. Mol. Cell **49**, 18–29. (10.1016/j.molcel.2012.10.015) 23177736 PMC3646334

[76] Barraud P, Gato A, Heiss M, Catala M, Kellner S, Tisné C. 2019 Time-resolved NMR monitoring of tRNA maturation. Nat. Commun. **10**, 3373. (10.1038/s41467-019-11356-w) 31358763 PMC6662845

[77] Masuda I, Takase R, Matsubara R, Paulines MJ, Gamper H, Limbach PA, Hou YM. 2018 Selective terminal methylation of a tRNA wobble base. Nucleic Acids Res. **46**, e37–e37. (10.1093/nar/gky013) 29361055 PMC5909439

[78] Charette M, Gray MW. 2000 Pseudouridine in RNA: what, where, how, and why. IUBMB Life **49**, 341–351. (10.1080/152165400410182) 10902565

[79] Kinghorn SM, O’Byrne CP, Booth IR, Stansfield I. 2002 Physiological analysis of the role of truB in Escherichia coli: a role for tRNA modification in extreme temperature resistance. Microbiology **148**, 3511–3520. (10.1099/00221287-148-11-3511) 12427942

[80] Ishida K, Kunibayashi T, Tomikawa C, Ochi A, Kanai T, Hirata A, Iwashita C, Hori H. 2011 Pseudouridine at position 55 in tRNA controls the contents of other modified nucleotides for low-temperature adaptation in the extreme-thermophilic eubacterium Thermus thermophilus. Nucleic Acids Res. **39**, 2304–2318. (10.1093/nar/gkq1180) 21097467 PMC3064792

[81] Dalluge JJ, Hashizume T, Sopchik AE, McCloskey JA, Davis DR. 1996 Conformational flexibility in RNA: the role of dihydrouridine. Nucleic Acids Res. **24**, 1073–1079. (10.1093/nar/24.6.1073) 8604341 PMC145759

[82] Alexandrov A, Chernyakov I, Gu W, Hiley SL, Hughes TR, Grayhack EJ, Phizicky EM. 2006 Rapid tRNA decay can result from lack of nonessential modifications. Mol. Cell **21**, 87–96. (10.1016/j.molcel.2005.10.036) 16387656

[83] Bacusmo JM *et al*. 2018 The t(6)A modification acts as a positive determinant for the anticodon nuclease PrrC, and is distinctively nonessential in Streptococcus mutans. RNA Biol. **15**, 508–517. (10.1080/15476286.2017.1353861) 28726545 PMC6103680

[84] Crick FH. 1966 Codon–anticodon pairing: the wobble hypothesis. J. Mol. Biol. **19**, 548–555. (10.1016/s0022-2836(66)80022-0) 5969078

[85] Agris PF, Vendeix FAP, Graham WD. 2007 tRNA’s wobble decoding of the genome: 40 years of modification. J. Mol. Biol. **366**, 1–13. (10.1016/j.jmb.2006.11.046) 17187822

[86] Meier F, Suter B, Grosjean H, Keith G, Kubli E. 1985 Queuosine modification of the wobble base in tRNAHis influences ‘in vivo’ decoding properties. EMBO J. **4**, 823–827. (10.1002/j.1460-2075.1985.tb03704.x) 2988936 PMC554263

[87] Fruchard L *et al*. 2025 Aminoglycoside tolerance in Vibrio cholerae engages translational reprogramming associated with queuosine tRNA modification. elife **13**, RP96317. (10.7554/eLife.96317) 39761105 PMC11703503

[88] Ehrenhofer-Murray AE. 2025 Queuine: a bacterial nucleobase shaping translation in eukaryotes. J. Mol. Biol. **437**, 168985. (10.1016/j.jmb.2025.168985) 39956693

[89] Nasvall SJ, Chen P, Björk GR. 2007 The wobble hypothesis revisited: uridine-5-oxyacetic acid is critical for reading of G-ending codons. RNA **13**, 2151–2164. (10.1261/rna.731007) 17942742 PMC2080601

[90] Sekiya T, Takeishi K, Ukita T. 1969 Specificity of yeast glutamic acid transfer RNA for codon recognition. Biochim. Biophys. Acta **182**, 411–426. (10.1016/0005-2787(69)90192-0) 4894016

[91] Nilsson EM, Alexander RW. 2019 Bacterial wobble modifications of NNA‐decoding tRNAs. IUBMB Life **71**, 1158–1166. (10.1002/iub.2120) 31283100 PMC6893868

[92] Yarus M. 1982 Translational efficiency of transfer RNA’s: uses of an extended anticodon. Science **218**, 646–652. (10.1126/science.6753149) 6753149

[93] Vendeix FAP, Dziergowska A, Gustilo EM, Graham WD, Sproat B, Malkiewicz A, Agris PF. 2008 Anticodon domain modifications contribute order to tRNA for ribosome-mediated codon binding. Biochemistry **47**, 6117–6129. (10.1021/bi702356j) 18473483

[94] Hou YM, Masuda I, Gamper H. 2018 Codon-specific translation by m1G37 methylation of tRNA. Front. Genet. **9**, 713. (10.3389/fgene.2018.00713) 30687389 PMC6335274

[95] Masuda I *et al*. 2022 tRNA methylation resolves codon usage bias at the limit of cell viability. Cell Rep. **41**, 111539. (10.1016/j.celrep.2022.111539) 36288695 PMC9643105

[96] Aubee JI, Olu M, Thompson KM. 2016 The i6A37 tRNA modification is essential for proper decoding of UUX-Leucine codons during rpoS and iraP translation. RNA **22**, 729–742. (10.1261/rna.053165.115) 26979278 PMC4836647

[97] Agris PF. 2008 Bringing order to translation: the contributions of transfer RNA anticodon‐domain modifications. EMBO Rep. **9**, 629–635. (10.1038/embor.2008.104) 18552770 PMC2475317

[98] Frye M, Jaffrey SR, Pan T, Rechavi G, Suzuki T. 2016 RNA modifications: what have we learned and where are we headed? Nat. Rev. Genet. **17**, 365–372. (10.1038/nrg.2016.47) 27140282

[99] Sarkar A, Gasperi W, Begley U, Nevins S, Huber SM, Dedon PC, Begley TJ. 2021 Detecting the epitranscriptome. Wiley Interdiscip. Rev. RNA **12**, e1663. (10.1002/wrna.1663) 33987958

[100] Gingold H, Pilpel Y. 2011 Determinants of translation efficiency and accuracy. Mol. Syst. Biol. **7**, 481. (10.1038/msb.2011.14) 21487400 PMC3101949

[101] Endres L, Dedon PC, Begley TJ. 2015 Codon-biased translation can be regulated by wobble-base tRNA modification systems during cellular stress responses. RNA Biol. **12**, 603–614. (10.1080/15476286.2015.1031947) 25892531 PMC4615639

[102] Dedon PC, Begley TJ. 2014 A System of RNA modifications and biased codon use controls cellular stress response at the level of translation. Chem. Res. Toxicol. **27**, 330–337. (10.1021/tx400438d) 24422464 PMC3997223

[103] Díaz-Rullo J, González-Pastor JE. 2023 tRNA queuosine modification is involved in biofilm formation and virulence in bacteria. Nucleic Acids Res. **51**, 9821–9837. (10.1093/nar/gkad667) 37638766 PMC10570037

[104] Thongdee N *et al*. 2019 TrmB, a tRNA m7G46 methyltransferase, plays a role in hydrogen peroxide resistance and positively modulates the translation of katA and katB mRNAs in Pseudomonas aeruginosa. Nucleic Acids Res. **47**, 9271–9281. (10.1093/nar/gkz702) 31428787 PMC6755087

[105] Gall AR, Datsenko KA, Figueroa-Bossi N, Bossi L, Masuda I, Hou YM, Csonka LN. 2016 Mg2+ regulates transcription of mgtA in Salmonella typhimurium via translation of proline codons during synthesis of the MgtL peptide. Proc. Natl Acad. Sci. USA **113**, 15096–15101. (10.1073/pnas.1612268113) 27849575 PMC5206563

[106] Chevance FFV, Hughes KT. 2017 Case for the genetic code as a triplet of triplets. Proc. Natl Acad. Sci. USA **114**, 4745–4750. (10.1073/pnas.1614896114) 28416671 PMC5422812

[107] Gingold H, Dahan O, Pilpel Y. 2012 Dynamic changes in translational efficiency are deduced from codon usage of the transcriptome. Nucleic Acids Res. **40**, 10053–10063. (10.1093/nar/gks772) 22941644 PMC3488229

[108] Balaban NQ *et al*. 2019 Definitions and guidelines for research on antibiotic persistence. Nat. Rev. Microbiol. **17**, 441–448. (10.1038/s41579-019-0196-3) 30980069 PMC7136161

[109] Brauner A, Fridman O, Gefen O, Balaban NQ. 2016 Distinguishing between resistance, tolerance and persistence to antibiotic treatment. Nat. Rev. Microbiol. **14**, 320–330. (10.1038/nrmicro.2016.34) 27080241

[110] Deventer AT, Stevens CE, Stewart A, Hobbs JK. 2024 Antibiotic tolerance among clinical isolates: mechanisms, detection, prevalence, and significance. Clin. Microbiol. Rev. **37**, e0010624. (10.1128/cmr.00106-24) 39364999 PMC11629620

[111] Toh SM, Mankin AS. 2008 An indigenous posttranscriptional modification in the ribosomal peptidyl transferase center confers resistance to an array of protein synthesis inhibitors. J. Mol. Biol. **380**, 593–597. (10.1016/j.jmb.2008.05.027) 18554609 PMC5367387

[112] Babosan A, Fruchard L, Krin E, Carvalho A, Mazel D, Baharoglu Z. 2022 Nonessential tRNA and rRNA modifications impact the bacterial response to sub-MIC antibiotic stress. microLife **3**, uqac019. (10.1093/femsml/uqac019) 37223353 PMC10117853

[113] Masuda I *et al*. 2019 tRNA methylation is a global determinant of bacterial multi-drug resistance. Cell Syst. **8**, 302–314. (10.1016/j.cels.2019.03.008) 30981730 PMC6483872

[114] Masuda I, Hwang JY, Christian T, Maharjan S, Mohammad F, Gamper H, Buskirk AR, Hou YM. 2021 Loss of N1-methylation of G37 in tRNA induces ribosome stalling and reprograms gene expression. Elife **10**, e70619. (10.7554/elife.70619) 34382933 PMC8384417

[115] Alvarez-Manzo HS *et al*. 2022 Yersinia pseudotuberculosis doxycycline tolerance strategies include modulating expression of genes involved in cell permeability and tRNA modifications. PLoS Pathog. **18**, e1010556. (10.1371/journal.ppat.1010556) 35576231 PMC9135342

[116] Pollo-Oliveira L *et al*. 2022 The absence of the queuosine tRNA modification leads to pleiotropic phenotypes revealing perturbations of metal and oxidative stress homeostasis in Escherichia coli K12. Metallomics **14**, mfac065. (10.1093/mtomcs/mfac065) 36066904 PMC9508795

[117] Schultz SK, Meadows K, Kothe U. 2023 Molecular mechanism of tRNA binding by the Escherichia coli N7 guanosine methyltransferase TrmB. J. Biol. Chem. **299**, 104612. (10.1016/j.jbc.2023.104612) 36933808 PMC10130221

[118] McGuffey JC *et al*. 2023 The tRNA methyltransferase TrmB is critical for Acinetobacter baumannii stress responses and pulmonary infection. mBio **14**, e0141623. (10.1128/mbio.01416-23) 37589464 PMC10653896

[119] Hua X, Chen Q, Li X, Yu Y. 2014 Global transcriptional response of Acinetobacter baumannii to a subinhibitory concentration of tigecycline. Int. J. Antimicrob. Agents **44**, 337–344. (10.1016/j.ijantimicag.2014.06.015) 25176631

[120] Chen Q, Li X, Zhou H, Jiang Y, Chen Y, Hua X, Yu Y. 2014 Decreased susceptibility to tigecycline in Acinetobacter baumannii mediated by a mutation in trm encoding SAM-dependent methyltransferase. J. Antimicrob. Chemother. **69**, 72–76. (10.1093/jac/dkt319) 23928024

[121] Bougdour A, Wickner S, Gottesman S. 2006 Modulating RssB activity: IraP, a novel regulator of sigma(S) stability in Escherichia coli. Genes Dev. **20**, 884–897. (10.1101/gad.1400306) 16600914 PMC1472289

[122] Thompson KM, Gottesman S. 2014 The MiaA tRNA modification enzyme is necessary for robust RpoS expression in Escherichia coli. J. Bacteriol. **196**, 754–761. (10.1128/jb.01013-13) 24296670 PMC3911166

[123] Aubee J, Olu M, Thompson K. 2017 TrmL and TusA are necessary for rpoS and MiaA is required for hfq expression in Escherichia coli. Biomolecules **7**, 39. (10.3390/biom7020039) 28471404 PMC5485728

[124] Aubee JI, Williams K, Adigun A, Olusanya O, Nurse J, Thompson KM. 2024 Post-transcriptional regulation of the MiaA prenyl transferase by CsrA and the small RNA CsrB in E. coli. BioRxiv 2024.02.28.582573. (10.1101/2024.02.28.582573) PMC12249760 40649849

[125] Koshla O *et al*. 2019 Gene miaA for post-transcriptional modification of tRNA(XXA) is important for morphological and metabolic differentiation in Streptomyces. Mol. Microbiol. **112**, 249–265. (10.1111/mmi.14266) 31017319

[126] Tomasi FG, Kimura S, Rubin EJ, Waldor MK. 2023 A tRNA modification in Mycobacterium tuberculosis facilitates optimal intracellular growth. Elife **12**, RP87146. (10.7554/eLife.87146.3) 37755167 PMC10531406

[127] Zhou J *et al*. 2021 Iron–sulfur biology invades tRNA modification: the case of U34 sulfuration. Nucleic Acids Res. **49**, 3997–4007. (10.1093/nar/gkab138) 33744947 PMC8053098

[128] Durand JM, Dagberg B, Uhlin BE, Bjork GR. 2000 Transfer RNA modification, temperature and DNA superhelicity have a common target in the regulatory network of the virulence of Shigella flexneri: the expression of the virF gene. Mol. Microbiol. **35**, 924–935. (10.1046/j.1365-2958.2000.01767.x) 10692168

[129] Shippy D, Fadl AA. 2014 tRNA modification enzymes GidA and MnmE: potential role in virulence of bacterial pathogens. Int. J. Mol. Sci. **15**, 18267–18280. (10.3390/ijms151018267) 25310651 PMC4227215

[130] Krueger J *et al*. 2024 tRNA epitranscriptome determines pathogenicity of the opportunistic pathogen Pseudomonas aeruginosa. Proc. Natl Acad. Sci. USA **121**, e2312874121. (10.1073/pnas.2312874121) 38451943 PMC10945773

[131] Rimbach K, Kaiser S, Helm M, Dalpke AH, Eigenbrod T. 2015 2’-O-Methylation within bacterial RNA Acts as suppressor of TLR7/TLR8 activation in human innate immune cells. J. Innate Immun. **7**, 482–493. (10.1159/000375460) 25823462 PMC6738864

[132] Galvanin A *et al*. 2020 Bacterial tRNA 2′-O-methylation is dynamically regulated under stress conditions and modulates innate immune response. Nucleic Acids Res. **48**, 12833–12844. (10.1093/nar/gkaa1123) 33275131 PMC7736821

[133] Vecerek B, Moll I, Bläsi U. 2007 Control of Fur synthesis by the non-coding RNA RyhB and iron-responsive decoding. EMBO J. **26**, 965–975. (10.1038/sj.emboj.7601553) 17268550 PMC1852835

[134] Lee WL *et al*. 2023 An RNA modification enzyme directly senses reactive oxygen species for translational regulation in Enterococcus faecalis. Nat. Commun. **14**, 4093. (10.1038/s41467-023-39790-x) 37433804 PMC10336011

[135] Kilz LM, Zimmermann S, Marchand V, Bourguignon V, Sudol C, Brégeon D, Hamdane D, Motorin Y, Helm M. 2024 Differential redox sensitivity of tRNA dihydrouridylation. Nucleic Acids Res. **52**, 12784–12797. (10.1093/nar/gkae964) 39460624 PMC11602153

[136] Shippy DC, Eakley NM, Bochsler PN, Chopra AK, Fadl AA. 2011 Biological and virulence characteristics of Salmonella enterica serovar Typhimurium following deletion of glucose-inhibited division (gidA) gene. Microb. Pathog. **50**, 303–313. (10.1016/j.micpath.2011.02.004) 21320585

[137] Jaroensuk J *et al*. 2016 Methylation at position 32 of tRNA catalyzed by TrmJ alters oxidative stress response in Pseudomonas aeruginosa. Nucleic Acids Res. **44**, 10834–10848. (10.1093/nar/gkw870) 27683218 PMC5159551

[138] Sato Y, Takita A, Suzue K, Hashimoto Y, Hiramoto S, Murakami M, Tomita H, Hirakawa H. 2024 TusDCB, a sulfur transferase complex involved in tRNA modification, contributes to UPEC pathogenicity. Sci. Rep. **14**, 8978. (10.1038/s41598-024-59614-2) 38637685 PMC11026471

[139] de Crecy-Lagard V *et al*. 2025 Are bacterial processes dependent on global ribosome pausing affected by tRNA modification defects? J. Mol. Biol. **437**, 169107. (10.1016/j.jmb.2025.169107) 40210524 PMC12162200

[140] Kopietz K *et al*. 2025 TGT damages its substrate tRNAs by the formation of abasic sites in the anticodon loop. J. Mol. Biol. **437**, 169000. (10.1016/j.jmb.2025.169000) 40011082

[141] Valesyan S, Jora M, Addepalli B, Limbach PA. 2024 Stress-induced modification of Escherichia coli tRNA generates 5-methylcytidine in the variable loop. Proc. Natl Acad. Sci. USA **121**, e2317857121. (10.1073/pnas.2317857121) 39495928 PMC11572931

[142] Sun C, Jora M, Solivio B, Limbach PA, Addepalli B. 2018 The effects of ultraviolet radiation on nucleoside modifications in RNA. ACS Chem. Biol. **13**, 567–572. (10.1021/acschembio.7b00898) 29400945 PMC5933051

[143] Thomas G, Favre A. 1975 4-Thiouridine as the target for near-ultraviolet light induced growth delay in Escherichia coli. Biochem. Biophys. Res. Commun. **66**, 1454–1461. (10.1016/0006-291x(75)90522-7) 1103892

[144] Bishop AC, Xu J, Johnson RC, Schimmel P, de Crécy-Lagard V. 2002 Identification of the tRNA-dihydrouridine synthase family. J. Biol. Chem. **277**, 25090–25095. (10.1074/jbc.m203208200) 11983710

[145] Brégeon D *et al*. 2022 Dihydrouridine in the transcriptome: new life for this ancient RNA chemical modification. ACS Chem. Biol. **17**, 1638–1657. (10.1021/acschembio.2c00307) 35737906

[146] Cho KH, Caparon MG. 2008 tRNA modification by GidA/MnmE is necessary for Streptococcus pyogenes virulence: a new strategy to make live attenuated strains. Infect. Immun. **76**, 3176–3186. (10.1128/iai.01721-07) 18426891 PMC2446735

[147] Görlitz K, Bessler L, Helm M, Schaffrath R, Klassen R. 2024 Fluoropyrimidines trigger decay of hypomodified tRNA in yeast. Nucleic Acids Res. **52**, 5841–5851. (10.1093/nar/gkae341) 38716877 PMC11162795

[148] Raina M, Ibba M. 2014 tRNAs as regulators of biological processes. Front. Genet. **5**, 171. (10.3389/fgene.2014.00171) 24966867 PMC4052509

[149] Borek E, Baliga BS, Gehrke CW, Kuo CW, Belman S, Troll W, Waalkes TP. 1977 High turnover rate of transfer RNA in tumor tissue. Cancer Res. **37**, 3362–3366. 884680

[150] Levitz R, Chapman D, Amitsur M, Green R, Snyder L, Kaufmann G. 1990 The optional E. coli prr locus encodes a latent form of phage T4-induced anticodon nuclease. EMBO J. **9**, 1383–1389. (10.1002/j.1460-2075.1990.tb08253.x) 1691706 PMC551823

[151] Gebetsberger J, Wyss L, Mleczko AM, Reuther J, Polacek N. 2017 A tRNA-derived fragment competes with mRNA for ribosome binding and regulates translation during stress. RNA Biol. **14**, 1364–1373. (10.1080/15476286.2016.1257470) 27892771 PMC5711459

[152] Li Z, Stanton BA. 2021 Transfer RNA-derived fragments, the underappreciated regulatory small RNAs in microbial pathogenesis. Front. Microbiol. **12**, 687632. (10.3389/fmicb.2021.687632) 34079534 PMC8166272

[153] Winther K, Tree JJ, Tollervey D, Gerdes K. 2016 VapCs of Mycobacterium tuberculosis cleave RNAs essential for translation. Nucleic Acids Res. **44**, 9860–9871. (10.1093/nar/gkw781) 27599842 PMC5175351

[154] Guzzi N *et al*. 2018 Pseudouridylation of tRNA-derived fragments steers translational control in stem cells. Cell **173**, 1204–1216. (10.1016/j.cell.2018.03.008) 29628141

[155] Frye M, Blanco S. 2016 Post-transcriptional modifications in development and stem cells. Development **143**, 3871–3881. (10.1242/dev.136556) 27803056

[156] Lyons SM, Fay MM, Ivanov P. 2018 The role of RNA modifications in the regulation of tRNA cleavage. FEBS Lett. **592**, 2828–2844. (10.1002/1873-3468.13205) 30058219 PMC6986807

[157] Helm M, Alfonzo JD. 2014 Posttranscriptional RNA modifications: playing metabolic games in a cell’s chemical legoland. Chem. Biol. **21**, 174–185. (10.1016/j.chembiol.2013.10.015) 24315934 PMC3944000

[158] Shepherd J, Ibba M. 2013 Direction of aminoacylated transfer RNAs into antibiotic synthesis and peptidoglycan‐mediated antibiotic resistance. FEBS Lett. **587**, 2895–2904. (10.1016/j.febslet.2013.07.036) 23907010 PMC3786784

[159] Lloyd AJ *et al*. 2008 Characterization of tRNA-dependent peptide bond formation by MurM in the synthesis of Streptococcus pneumoniae peptidoglycan. J. Biol. Chem. **283**, 6402–6417. (10.1074/jbc.m708105200) 18077448

[160] Mogk A, Schmidt R, Bukau B. 2007 The N-end rule pathway for regulated proteolysis: prokaryotic and eukaryotic strategies. Trends Cell Biol. **17**, 165–172. (10.1016/j.tcb.2007.02.001) 17306546

[161] Gutgsell N, Englund N, Niu L, Kaya Y, Lane BG, Ofengand J. 2000 Deletion of the Escherichia coli pseudouridine synthase gene truB blocks formation of pseudouridine 55 in tRNA in vivo, does not affect exponential growth, but confers a strong selective disadvantage in competition with wild-type cells. RNA **6**, 1870–1881. (10.1017/s1355838200001588) 11142385 PMC1370055

[162] Benítez-Páez A, Villarroya M, Armengod ME. 2012 The Escherichia coli RlmN methyltransferase is a dual-specificity enzyme that modifies both rRNA and tRNA and controls translational accuracy. RNA **18**, 1783–1795. (10.1261/rna.033266.112) 22891362 PMC3446703

[163] Addepalli B, Limbach PA. 2016 Pseudouridine in the anticodon of Escherichia coli tRNATyr(QΨA) is catalyzed by the dual specificity enzyme RluF. J. Biol. Chem. **291**, 22327–22337. (10.1074/jbc.m116.747865) 27551044 PMC5064010

[164] Wrzesinski J, Nurse K, Bakin A, Lane BG, Ofengand J. 1995 A dual-specificity pseudouridine synthase: an Escherichia coli synthase purified and cloned on the basis of its specificity for psi 746 in 23S RNA is also specific for psi 32 in tRNA(phe). RNA **1**, 437–448. 7493321 PMC1482406

[165] Schaening-Burgos C, LeBlanc H, Fagre C, Li GW, Gilbert WV. 2024 RluA is the major mRNA pseudouridine synthase in Escherichia coli. PLoS Genet. **20**, e1011100. (10.1371/journal.pgen.1011100) 39241085 PMC11421799

[166] Lin Q, Huang J, Liu Z, Chen Q, Wang X, Yu G, Cheng P, Zhang LH, Xu Z. 2022 tRNA modification enzyme MiaB connects environmental cues to activation of Pseudomonas aeruginosa type III secretion system. PLoS Pathog. **18**, e1011027. (10.1371/journal.ppat.1011027) 36469533 PMC9754610

[167] Adeleye SA, Yadavalli SS. 2024 Queuosine biosynthetic enzyme, QueE moonlights as a cell division regulator. BioRxiv (10.1101/2023.10.31.565030) PMC11142719 38768229

[168] Muraski MJ, Nilsson EM, Fritz MJ, Richardson AR, Alexander RW, Cooper VS. 2023 Adaptation to overflow metabolism by mutations that impair tRNA modification in experimentally evolved bacteria. mBio **14**, e0028723. (10.1128/mbio.00287-23) 36853041 PMC10128029

[169] Phizicky EM, Hopper AK. 2010 tRNA biology charges to the front. Genes Dev. **24**, 1832–1860. (10.1101/gad.1956510) 20810645 PMC2932967

[170] Herschlag D. 1995 RNA chaperones and the RNA folding problem. J. Biol. Chem. **270**, 20871–20874. (10.1074/jbc.270.36.20871) 7545662

[171] Woodson SA. 2010 Taming free energy landscapes with RNA chaperones. RNA Biol. **7**, 677–686. (10.4161/rna.7.6.13615) 21045544 PMC3073327

[172] Treiber DK, Williamson JR. 1999 Exposing the kinetic traps in RNA folding. Curr. Opin. Struct. Biol. **9**, 339–345. (10.1016/s0959-440x(99)80045-1) 10361090

[173] Russell R, Zhuang X, Babcock HP, Millett IS, Doniach S, Chu S, Herschlag D. 2002 Exploring the folding landscape of a structured RNA. Proc. Natl Acad. Sci. USA **99**, 155–160. (10.1073/pnas.221593598) 11756689 PMC117531

[174] Solomatin SV, Greenfeld M, Chu S, Herschlag D. 2010 Multiple native states reveal persistent ruggedness of an RNA folding landscape. Nature **463**, 681–684. (10.1038/nature08717) 20130651 PMC2818749

[175] Thirumalai D, Hyeon C. 2005 RNA and protein folding: common themes and variations. Biochemistry **44**, 4957–4970. (10.1021/bi047314+) 15794634

[176] Weeks KM. 1997 Protein-facilitated RNA folding. Curr. Opin. Struct. Biol. **7**, 336–342. (10.1016/s0959-440x(97)80048-6) 9204274

[177] Godet J, Boudier C, Humbert N, Ivanyi-Nagy R, Darlix JL, Mély Y. 2012 Comparative nucleic acid chaperone properties of the nucleocapsid protein NCp7 and Tat protein of HIV-1. Virus Res. **169**, 349–360. (10.1016/j.virusres.2012.06.021) 22743066 PMC7114403

[178] Porat J, Kothe U, Bayfield MA. 2021 Revisiting tRNA chaperones: new players in an ancient game. RNA **27**, 543–559. (10.1261/rna.078428.120) 33593999 PMC8051267

[179] Maraia RJ, Mattijssen S, Cruz-Gallardo I, Conte MR. 2017 The La and related RNA-binding proteins (LARPs): structures, functions, and evolving perspectives. Wiley Interdiscip. Rev. RNA **8**. (10.1002/wrna.1430) PMC5647580 28782243

[180] Keffer-Wilkes LC, Veerareddygari GR, Kothe U. 2016 RNA modification enzyme TruB is a tRNA chaperone. Proc. Natl Acad. Sci. USA **113**, 14306–14311. (10.1073/pnas.1607512113) 27849601 PMC5167154

[181] Keffer-Wilkes LC, Soon EF, Kothe U. 2020 The methyltransferase TrmA facilitates tRNA folding through interaction with its RNA-binding domain. Nucleic Acids Res. **48**, 7981–7990. (10.1093/nar/gkaa548) 32597953 PMC7641329

[182] Alian A, Lee TT, Griner SL, Stroud RM, Finer-Moore J. 2008 Structure of a TrmA-RNA complex: a consensus RNA fold contributes to substrate selectivity and catalysis in m5U methyltransferases. Proc. Natl Acad. Sci. USA **105**, 6876–6881. (10.1073/pnas.0802247105) 18451029 PMC2383949

[183] Jones JD *et al*. 2024 Conserved 5-methyluridine tRNA modification modulates ribosome translocation. Proc. Natl Acad. Sci. USA **121**, e2401743121. (10.1073/pnas.2401743121) 39159370 PMC11363252

[184] Rajkowitsch L *et al*. 2007 RNA chaperones, RNA annealers and RNA helicases. RNA Biol. **4**, 118–130. (10.4161/rna.4.3.5445) 18347437

[185] Bose D, Chakrabarti A. 2017 Substrate specificity in the context of molecular chaperones. IUBMB Life **69**, 647–659. (10.1002/iub.1656) 28748601

[186] Yu F, Tanaka Y, Yamashita K, Suzuki T, Nakamura A, Hirano N, Suzuki T, Yao M, Tanaka I. 2011 Molecular basis of dihydrouridine formation on tRNA. Proc. Natl Acad. Sci. USA **108**, 19593–19598. (10.1073/pnas.1112352108) 22123979 PMC3241823

[187] Hoang C, Ferré-D’Amaré AR. 2001 Cocrystal structure of a tRNA Psi55 pseudouridine synthase: nucleotide flipping by an RNA-modifying enzyme. Cell **107**, 929–939. (10.1016/s0092-8674(01)00618-3) 11779468

[188] Finer-Moore J, Czudnochowski N, O’Connell JD, Wang AL, Stroud RM. 2015 Crystal structure of the human tRNA m1A58 methyltransferase–tRNA3Lys complex: refolding of substrate tRNA allows access to the methylation target. J. Mol. Biol. **427**, 3862–3876. (10.1016/j.jmb.2015.10.005) 26470919 PMC4663122

[189] Gc K, Gyawali P, Balci H, Abeysirigunawardena S. 2020 Ribosomal RNA methyltransferase RsmC moonlights as an RNA chaperone. ChemBioChem **21**, 1885–1892. (10.1002/cbic.201900708) 31972066

[190] Bjork GR, Hagervall TG. 2014 Transfer RNA modification: presence, synthesis, and function. EcoSal Plus **6**, ESP-0007-2013. (10.1128/ecosalplus.ESP-0007-2013) 26442937

[191] de Crecy‐Lagard V, Marck C, Brochier‐Armanet C, Grosjean H. 2007 Comparative RNomics and modomics in mollicutes: prediction of gene function and evolutionary implications. IUBMB Life **59**, 634–658. (10.1080/15216540701604632) 17852564

[192] de Crecy-Lagard V, Ross R, Jaroch M, Marchand V, Eisenhart C, Bregeon D, Motorin Y, Limbach P. 2020 Survey and validation of tRNA modifications and their corresponding genes in Bacillus subtilis sp. subtilis strain 168. Biomolecules **10**, 977. (10.3390/biom10070977) 32629984 PMC7408541

[193] Mandler MD, Maligireddy SS, Guiblet WM, Fitzsimmons CM, McDonald KS, Warrell DL, Batista PJ. 2024 The modification landscape of Pseudomonas aeruginosa tRNAs. RNA **30**, 1025–1040. (10.1261/rna.080004.124) 38684317 PMC11251520

[194] Quaiyum S, Sun J, Marchand V, Sun G, Reed CJ, Motorin Y, Dedon PC, Minnick MF, de Crécy-Lagard V. 2024 Mapping the tRNA modification landscape of Bartonella henselae Houston I and Bartonella quintana Toulouse. Front. Microbiol. **15**, 1369018. (10.3389/fmicb.2024.1369018) 38544857 PMC10965804

[195] Antoine L, Wolff P, Westhof E, Romby P, Marzi S. 2019 Mapping post-transcriptional modifications in Staphylococcus aureus tRNAs by nanoLC/MSMS. Biochimie **164**, 60–69. (10.1016/j.biochi.2019.07.003) 31295507

[196] Yu N, Jora M, Solivio B, Thakur P, Acevedo-Rocha CG, Randau L, de Crécy-Lagard V, Addepalli B, Limbach PA. 2019 tRNA modification profiles and codon-decoding strategies in Methanocaldococcus jannaschii. J. Bacteriol. **201**, e00690-18. (10.1128/JB.00690-18) 30745370 PMC6456858

[197] Koshla O, Vogt LM, Rydkin O, Sehin Y, Ostash I, Helm M, Ostash B. 2023 Landscape of post-transcriptional tRNA modifications in Streptomyces albidoflavus J1074 as portrayed by mass spectrometry and genomic data mining. J. Bacteriol. **205**, e0029422. (10.1128/jb.00294-22) 36468867 PMC9879100

[198] Panosyan H, Traube FR, Brandmayr C, Wagner M, Carell T. 2022 tRNA modification profiles in obligate and moderate thermophilic bacilli. Extremophiles **26**, 11. (10.1007/s00792-022-01258-z) 35122547 PMC8818000

[199] Miyauchi K *et al*. 2024 A tRNA modification with aminovaleramide facilitates AUA decoding in protein synthesis. Nat. Chem. Biol. **21**, 522–531. (10.1038/s41589-024-01726-x) 39300229 PMC11938285

[200] Kimura S, Dedon PC, Waldor MK. 2020 Comparative tRNA sequencing and RNA mass spectrometry for surveying tRNA modifications. Nat. Chem. Biol. **16**, 964–972. (10.1038/s41589-020-0558-1) 32514182 PMC8172280

[201] Urbonavicius J, Skouloubris S, Myllykallio H, Grosjean H. 2005 Identification of a novel gene encoding a flavin-dependent tRNA:m5U methyltransferase in bacteria—evolutionary implications. Nucleic Acids Res. **33**, 3955–3964. (10.1093/nar/gki703) 16027442 PMC1178002

[202] Ryu H, Grove TL, Almo SC, Kim J. 2018 Identification of a novel tRNA wobble uridine modifying activity in the biosynthesis of 5-methoxyuridine. Nucleic Acids Res. **46**, 9160–9169. (10.1093/nar/gky592) 29982645 PMC6158493

[203] Cho G, Lee J, Kim J. 2023 Identification of a novel 5-aminomethyl-2-thiouridine methyltransferase in tRNA modification. Nucleic Acids Res. **51**, 1971–1983. (10.1093/nar/gkad048) 36762482 PMC9976899

[204] Kang B *et al*. 2017 Identification of 2-methylthio cyclic N6-threonylcarbamoyladenosine (ms2ct6A) as a novel RNA modification at position 37 of tRNAs. Nucleic Acids Res. **45**, 2124–2136. (10.1093/nar/gkw1120) 27913733 PMC5389704

[205] Sun J. 2024 tRNA modification profilingreveals epitranscriptome regulatory networks in Pseudomonas aeruginosa. bioRxiv 2024.07.01.601603. (10.1101/2024.07.01.601603)

[206] Zhang W, Foo M, Eren AM, Pan T. 2022 tRNA modification dynamics from individual organisms to metaepitranscriptomics of microbiomes. Mol. Cell **82**, 891–906. (10.1016/j.molcel.2021.12.007) 35032425 PMC8897278

[207] Yamagami R, Sieg JP, Assmann SM, Bevilacqua PC. 2022 Genome-wide analysis of the in vivo tRNA structurome reveals RNA structural and modification dynamics under heat stress. Proc. Natl Acad. Sci. USA **119**, e2201237119. (10.1073/pnas.2201237119) 35696576 PMC9231505

[208] Barrios SR, Camus LV, Cusack SA, Burdack K, Petrov DP, Yeşiltaç-Tosun GN, Kaiser S, Giehr P, Jung K. 2025 Direct RNA sequencing of the Escherichia coli epitranscriptome uncovers alterations under heat stress. Nucleic Acids Res. **53**, gkaf175. (10.1093/nar/gkaf175) 40114376 PMC11925731

[209] Hoffmann A, Lorenz C, Fallmann J, Wolff P, Lechner A, Betat H, Mörl M, Stadler PF. 2024 Temperature-dependent tRNA modifications in Bacillales. Int. J. Mol. Sci. **25**, 8823. (10.3390/ijms25168823) 39201508 PMC11354880

[210] Jaroch M, Savage K, Kuipers P, Bacusmo JM, Hu J, Sun J, Dedon PC, Rice KC, de Crécy-Lagard V. 2025 Environmental control of queuosine levels in Streptococcus mutans tRNAs. Mol. Microbiol. **123**, 48–59. (10.1111/mmi.15336) 39719891 PMC11724357

[211] Edwards AM, Black KA, Dos Santos PC. 2022 Sulfur availability impacts accumulation of the 2-thiouridine tRNA modification in Bacillus subtilis. J. Bacteriol. **204**, e0000922. (10.1128/jb.00009-22) 35467390 PMC9112905

[212] Zborowsky S, Tahan R, Lindell D. 2025 Adaptive loss of tRNA gene expression leads to phage resistance in a marine Synechococcus cyanobacterium. Nat. Microbiol. **10**, 66–76. (10.1038/s41564-024-01877-6) 39753669 PMC11726456

[213] Pozhydaieva N, Wolfram-Schauerte M, Keuthen H, Höfer K. 2024 The enigmatic epitranscriptome of bacteriophages: putative RNA modifications in viral infections. Curr. Opin. Microbiol. **77**, 102417. (10.1016/j.mib.2023.102417) 38217927

[214] Sintsova A, Frick-Cheng AE, Smith S, Pirani A, Subashchandrabose S, Snitkin ES, Mobley H. 2019 Genetically diverse uropathogenic Escherichia coli adopt a common transcriptional program in patients with UTIs. elife **8**, e49748. (10.7554/eLife.49748) 31633483 PMC6802966

[215] Fleming BA *et al*. 2022 A tRNA modifying enzyme as a tunable regulatory nexus for bacterial stress responses and virulence. Nucleic Acids Res. **50**, 7570–7590. (10.1093/nar/gkac116) 35212379 PMC9303304

[216] Shea AE, Marzoa J, Himpsl SD, Smith SN, Zhao L, Tran L, Mobley HLT. 2020 Escherichia coli CFT073 fitness factors during urinary tract infection: identification using an ordered transposon library. Appl. Environ. Microbiol. **86**, e00691-20. (10.1128/AEM.00691-20) 32358013 PMC7301846

[217] Chittrakanwong J *et al*. 2025 5-Methyluridine is ubiquitous in Pseudomonas aeruginosa tRNA and modulates antimicrobial resistance and virulence. J. Mol. Biol. **437**, 169020. (10.1016/j.jmb.2025.169020) 40055058 PMC12162239

[218] Frommeyer YN *et al*. 2025 tRNA hydroxylation is an epitranscriptomic modulator of metabolic states affecting Pseudomonas aeruginosa pathogenicity. Nucleic Acids Res. **53**, gkaf719. (10.1093/nar/gkaf719) 40705922 PMC12288880

[219] Fu Y, Waldor MK, Mekalanos JJ. 2013 Tn-Seq analysis of Vibrio cholerae intestinal colonization reveals a role for T6SS-mediated antibacterial activity in the host. Cell Host Microbe **14**, 652–663. (10.1016/j.chom.2013.11.001) 24331463 PMC3951154

[220] Kamp HD, Patimalla-Dipali B, Lazinski DW, Wallace-Gadsden F, Camilli A. 2013 Gene fitness landscapes of Vibrio cholerae at important stages of its life cycle. PLoS Pathog. **9**, e1003800. (10.1371/journal.ppat.1003800) 24385900 PMC3873450

[221] Di Martino ML *et al*. 2025 A scalable gut epithelial organoid model reveals the genome-wide colonization landscape of a human-adapted pathogen. Nat. Genet. **57**, 1730–1741. (10.1038/s41588-025-02218-x) 40506541 PMC12283395

[222] Morinière L, Mirabel L, Gueguen E, Bertolla F. 2022 A Comprehensive overview of the genes and functions required for lettuce infection by the hemibiotrophic phytopathogen Xanthomonas hortorum pv. vitians. mSystems **7**, e0129021. (10.1128/msystems.01290-21) 35311560 PMC9040725

[223] Bacusmo JM *et al*. 2024 Synergistic effects of tRNA modification defects in Escherichia coli K12. BioRxiv 2024.11.12.622971. (10.1101/2024.11.12.622971)

[224] Kompatscher M, Gonnella I, Erlacher M. 2025 Studying the function of tRNA modifications: experimental challenges and opportunities. J. Mol. Biol. **437**, 168934. (10.1016/j.jmb.2024.168934) 39756793

[225] White LK, Radakovic A, Sajek MP, Dobson K, Riemondy KA, Del Pozo S, Szostak JW, Hesselberth JR. 2025 Nanopore sequencing of intact aminoacylated tRNAs. Nat. Commun **16**, 7781. (10.1038/s41467-025-62545-9) 40835813 PMC12368100

[226] Delgado-Tejedor A, Medina R, Begik O, Cozzuto L, López J, Blanco S, Ponomarenko J, Novoa EM. 2024 Native RNA nanopore sequencing reveals antibiotic-induced loss of rRNA modifications in the A- and P-sites. Nat. Commun. **15**, 10054. (10.1038/s41467-024-54368-x) 39613750 PMC11607429

[227] Fleming AM, Xiao S, Burrows CJ. 2023 Nanopore sequencing for the 17 modification types in 36 locations in E. coli ribosomal RNA enables monitoring of stress-dependent changes. bioRxiv 2023.03.12.532289. (10.1101/2023.03.12.532289) PMC10594579 37345867

[228] Bahena-Ceron R *et al*. 2024 RlmQ: a newly discovered rRNA modification enzyme bridging RNA modification and virulence traits in Staphylococcus aureus. RNA **30**, 200–212. (10.1261/rna.079850.123) 38164596 PMC10870370

[229] National Academies of Sciences, Engineering, and Medicine; Health and Medicine Division; Division on Earth and Life Studies; Board on Health Sciences Policy; Board on Life Sciences; Toward Sequencing and Mapping of RNA Modifications Committee. 2024 Charting a future for sequencing RNA and its modifications: a new era for biology and medicine. Washington, DC: The National Academies Press. 39159274

[230] Zhong W *et al*. 2019 Targeting the bacterial epitranscriptome for antibiotic development: discovery of novel tRNA-(N(1)G37) methyltransferase (TrmD) Inhibitors. ACS Infect. Dis. **5**, 326–335. (10.1021/acsinfecdis.8b00275) 30682246

[231] Li N *et al*. 2021 METTL3 regulates viral m6A RNA modification and host cell innate immune responses during SARS-CoV-2 infection. Cell Rep. **35**, 109091. (10.1016/j.celrep.2021.109091) 33961823 PMC8090989

[232] Nance KD, Meier JL. 2021 Modifications in an emergency: the role of N1-methylpseudouridine in COVID-19 vaccines. ACS Cent. Sci. **7**, 748–756. (10.1021/acscentsci.1c00197) 34075344 PMC8043204

[233] Galvanin A, Ayadi L, Helm M, Motorin Y, Marchand V. 2019 Mapping and quantification of tRNA 2’-O-methylation by RiboMethSeq. Methods Mol. Biol. **1870**, 273–295. (10.1007/978-1-4939-8808-2_21) 30539563

[234] Marchand V. 2018 AlkAniline-Seq: profiling of m(7)G and m(3)C RNA modifications at single nucleotide resolution. Angew. Chem. **130**, 17027–17032. (10.1002/ange.201810946) 30370969

[235] Marchand V *et al*. 2020 HydraPsiSeq: a method for systematic and quantitative mapping of pseudouridines in RNA. Nucleic Acids Res. **48**, e110–e110. (10.1093/nar/gkaa769) 32976574 PMC7641733

[236] Marchand V, Pichot F, Thüring K, Ayadi L, Freund I, Dalpke A, Helm M, Motorin Y. 2017 Next‐generation sequencing‐based ribomethseq protocol for analysis of tRNA 2′‐O‐methylation. Biomolecules **7**, 13. (10.3390/biom7010013) 28208788 PMC5372725

[237] Katanski CD, Watkins CP, Zhang W, Reyer M, Miller S, Pan T. 2022 Analysis of queuosine and 2-thio tRNA modifications by high throughput sequencing. Nucleic Acids Res. **50**, e99–e99. (10.1093/nar/gkac517) 35713550 PMC9508811

[238] Zhong W *et al*. 2019 Thienopyrimidinone derivatives that inhibit bacterial tRNA (guanine37- N(1))-methyltransferase (TrmD) by restructuring the active site with a tyrosine-flipping mechanism. J. Med. Chem. **62**, 7788–7805. (10.1021/acs.jmedchem.9b00582) 31442049 PMC6748665

[239] Hill PJ *et al*. 2013 Selective inhibitors of bacterial t-RNA-(N(1)G37) methyltransferase (TrmD) that demonstrate novel ordering of the lid domain. J. Med. Chem. **56**, 7278–7288. (10.1021/jm400718n) 23981144

[240] Shapiro AB, Plant H, Walsh J, Sylvester M, Hu J, Gao N, Livchak S, Tentarelli S, Thresher J. 2014 Discovery of ATP-competitive inhibitors of tRNAIle lysidine synthetase (TilS) by high-throughput screening. J. Biomol. Screen. **19**, 1137–1146. (10.1177/1087057114534981) 24820111

[241] Kopina BJ, Missoury S, Collinet B, Fulton MG, Cirio C, van Tilbeurgh H, Lauhon CT. 2021 Structure of a reaction intermediate mimic in t6A biosynthesis bound in the active site of the TsaBD heterodimer from Escherichia coli. Nucleic Acids Res. **49**, 2141–2160. (10.1093/nar/gkab026) 33524148 PMC7913687

[242] Hou YM, Masuda I, Foster LJ. 2020 tRNA methylation: an unexpected link to bacterial resistance and persistence to antibiotics and beyond. Wiley Interdiscip. Rev. RNA **11**, e1609. (10.1002/wrna.1609) 32533808 PMC7768609

